# Optimizing Cognitive Training for the Treatment of Cognitive Dysfunction in Parkinson’s Disease: Current Limitations and Future Directions

**DOI:** 10.3389/fnagi.2021.709484

**Published:** 2021-10-13

**Authors:** Bianca Guglietti, David Hobbs, Lyndsey E. Collins-Praino

**Affiliations:** ^1^Cognition, Ageing and Neurodegenerative Disease Laboratory, Department of Medical Sciences, Adelaide Medical School, The University of Adelaide, Adelaide, SA, Australia; ^2^Medical Device Research Institute, College of Science and Engineering, Flinders University, Tonsley, SA, Australia; ^3^Allied Health & Human Performance, University of South Australia, Adelaide, SA, Australia

**Keywords:** dementia, mild cognitive impairment, neurorehabilitaiton, prevent, non-pharmaceutical, serious game, multimodal

## Abstract

Cognitive dysfunction, primarily involving impairments in executive function, visuospatial function and memory, is one of the most common non-motor symptoms of Parkinson’s disease (PD). Currently, the only pharmacological treatments available for the treatment of cognitive dysfunction in PD provide variable benefit, making the search for potential non-pharmacological therapies to improve cognitive function of significant interest. One such therapeutic strategy may be cognitive training (CT), which involves the repetition of standardized tasks with the aim of improving specific aspects of cognition. Several studies have examined the effects of CT in individuals with PD and have shown benefits in a variety of cognitive domains, but the widespread use of CT in these individuals may be limited by motor impairments and other concerns in study design. Here, we discuss the current state of the literature on the use of CT for PD and propose recommendations for future implementation. We also explore the potential use of more recent integrative, adaptive and assistive technologies, such as virtual reality, which may optimize the delivery of CT in PD.

## Introduction

In addition to the well-known motor impairments, Parkinson’s disease (PD) is also associated with significant cognitive dysfunction, manifesting primarily in five domains: executive function, attention, memory, speed of processing and visuospatial functioning (Williams-Gray et al., 2007; Kehagia et al., 2010). Importantly, individuals with PD carry six times the risk of dementia compared to the general population (Aarsland et al., 2001). Despite the prevalence of cognitive impairment in PD, however, pharmacological treatments have proven largely ineffective (Seppi et al., 2011). Thus, the use of non-pharmacological interventions to enhance cognitive function and to potentially prevent the emergence of dementia in PD is of significant importance.

This review will first briefly describe the prevalence and presentation of cognitive dysfunction in PD and discuss the literature regarding the effectiveness of cognitive training (CT). Although reviews and meta-analyses on CT in PD have previously been conducted (Calleo et al., 2012; Hindle et al., 2013; Leung et al., 2015; Biundo et al., 2017; Walton et al., 2017), the literature has since expanded. Additionally, significant variation in approaches to the implementation and evaluation of CT have complicated efforts to accurately assess the efficacy. This, coupled with the under-addressed need to tailor interventions for the PD population due to the unique involvement of motor symptoms, prompts the need for consideration of outcomes in the context of study design. This review will assess the evidence of current techniques to inform recommendations and provide insight into the potential utility of more recent integrative, adaptive and assistive technologies in order to optimize the delivery of CT in PD.

## PD and Cognitive Dysfunction

Cognitive impairment in PD ranges from mild cognitive impairment (PD-MCI) to Parkinson’s disease dementia (PD-D). Early on in PD, deficits are estimated to occur in 20–40% of individuals, although they are often overshadowed by motor features (Foltynie et al., 2004; Muslimovic et al., 2005; Elgh et al., 2009; Williams-Gray et al., 2009; Benito-Leon et al., 2011). These are usually subtle and detectable only with formal neuropsychological testing (Levin and Katzen, 2005) and include impairments on tests of immediate verbal recall, language production/semantic fluency, set formation, cognitive sequencing, working memory (WM), and visuomotor construction compared to healthy, non-demented controls (Cooper et al., 1991). In a population-based case-control study of individuals with early PD (<5 years duration), subjective memory complaints were present in 58.7% of PD patients, compared to 37% of controls (Benito-Leon et al., 2011). Similarly, a longitudinal population-based study of early PD patients revealed 30% were impaired in one or more cognitive domains (episodic memory, executive function and verbal function) (Elgh et al., 2009), indicating cognitive dysfunction may already be a source of considerable concern for PD patients at time of diagnosis.

Many individuals are also at increased risk of developing PD-MCI, with a meta-analysis involving over 1,346 PD patients indicating prevalence of MCI was 25.8% (Aarsland et al., 2010). At time of PD diagnosis, 15–20% of patients already meet criteria for PD-MCI (Aarsland, 2016) with this figure rising to 20–57% of individuals by 3–5 years post-diagnosis (Caviness et al., 2007; Williams-Gray et al., 2007). MCI in PD is an early stage of cognitive decline and clinical presentation of PD-MCI can be variable, ranging from amnestic to non-amnestic and single to multiple domains (Kalbe et al., 2016). However, of all cognitive functions, deficits in executive function (e.g., impairment in the ability to plan and to inhibit behaviors or deficits in attention and WM) are the most commonly seen in PD-MCI (Kalbe et al., 2016) and can severely impact an individual’s ability to carry out activities of daily living (ADL) (Pagonabarraga and Kulisevsky, 2012). These cognitive changes may be particularly tied to dopaminergic fronto-striatal function (Owen et al., 1992).

According to the “dual syndrome hypothesis,” while fronto-striatal executive deficits, common early in the course of PD, are more stable, cognitive deficits related to posterior cortical dysfunction, such as visuospatial function and recognition memory, are linked to earlier emergence of dementia in PD (Williams-Gray et al., 2007; Kehagia et al., 2013). A recent meta-analysis found 25% of PD patients with normal cognition convert to PD-MCI and 20% of PD-MCI patients convert to dementia within 3 years (Saredakis et al., 2019). Within 20 years of diagnosis, however, over 80% of individuals with PD progress to PD-D (Hely et al., 2008). In contrast to PD-MCI, PD-D is associated with more severe and debilitating cognitive impairments in multiple domains. Furthermore, PD-D includes both cognitive features (e.g., impairments in attention, executive function, visuospatial function and memory) and behavioral features (e.g., apathy, changes in personality and mood, hallucinations, delusions and excessive daytime sleepiness) (Emre et al., 2007). These neuropsychiatric and cognitive impairments are amongst the most debilitating for PD patients as non-motor symptoms account for the biggest predictors of quality of life (QoL), mortality and caregiver burden (Duncan et al., 2014).

### Pharmacological Treatment of Cognitive Impairment in PD: Limitations

Despite the prevalence of cognitive impairment in PD, its treatment has remained an area of unmet clinical need, with existing treatments providing only symptomatic relief of already-established dysfunction. Treatment of PD-D with dopaminergic strategies, such as L-dopa and dopamine agonists, has failed to produce significant improvements in cognition (Kulisevsky et al., 1996; Brusa et al., 2005; Akbar and Friedman, 2015). Treatments acting only on the dopaminergic system are likely to be ineffective, as PD-D involves alterations in a number of other neurotransmitter systems in addition to dopamine, such as noradrenaline, serotonin and acetylcholine (see Halliday et al., 2014 for review). In support of this, the norephinephrine reuptake inhibitor atomexitine has demonstrated mild benefits in PD patients with MCI in two small trials (Marsh et al., 2009; Weintraub et al., 2010). Furthermore, of all neurotransmitters altered in PD-D, cholinergic changes are most striking, with cortical cholinergic function more severely affected in PD-D than in Alzheimer’s disease (Hilker et al., 2005; Bohnen et al., 2006; Bohnen and Albin, 2011). In light of these abnormalities, the use of cholinesterase inhibitors is currently the preferred pharmacological treatment strategy for cognitive impairments in PD (Akbar and Friedman, 2015), and evidence from both large, randomized placebo-controlled trials and meta-analyses suggests that use of these compounds may provide at least modest benefit in the treatment of PD-D (Rolinski et al., 2012; Wang et al., 2015). A meta-analysis by Wang et al. (2015) found that both cholinesterase inhibitors and the NMDA receptor antagonist memantine provided a small benefit in PD-D and dementia with Lewy bodies, but only cholinesterase inhibitors led to a modest improvement in cognition as evaluated by MMSE (Wang et al., 2015).

Despite this, however, cholinesterase inhibitors have variable efficacy between patients, often not providing significant benefit (Emre et al., 2014). Furthermore, they may be associated with side-effects, such as gastrointestinal symptoms (Aarsland, 2016). Rivastigmine treatment has been linked to side-effects such as nausea (29%), vomiting (16.6%) and even tremor (10.2%) (Emre et al., 2014), leading to concerns long-term use of cholinesterase inhibitors could worsen motor symptoms in some PD patients. In support of this, both rivastigmine and donepezil can induce tremor in response to their administration in a subset of individuals (Gurevich et al., 2006; McCain et al., 2007; Song et al., 2008), and administration of the anticholinesterase galantamine can potently induce tremor (Collins et al., 2011). This highlights the need for a complimentary and/or alternate intervention strategy that may be adapted and targeted to address the specific needs of the individual.

## Cognitive Training in PD

Cognitive training (CT) is defined as training programs that provide structured practice on specific cognitive tasks, designed to improve performance in one or more cognitive domains, such as memory, attention or executive function (Clare and Woods, 2004). Although studies on the neural basis of CT in PD are sparse, and have included only a handful of subjects, studies in healthy aging may also provide insight. Systematic review of various CT and magnetic resonance imaging (MRI) modalities suggest structural changes in both gray and white matter occur following CT, particularly in the hippocampus (Belleville and Bherer, 2012; Valkanova et al., 2014). A subsequent MRI study has suggested these benefits may be due to neuroplasticity, as there was an increase in cerebral blood flow and neural connectivity in the default mode network and central executive network following CT (Chapman et al., 2015).

Extending this to PD specifically, CT may potentially lead to benefits by increasing neuroplasticity directly within the frontostriatal circuit, which is known to be compromised in PD. Compared to healthy controls, individuals with PD are known to have decreased functional connectivity within the fronto-striatal circuit (Xu et al., 2016). Such changes in functional connectivity within these circuits subsequently lead to many of the cognitive impairments that predominate in PD, including attention/memory, executive function and perception (recently reviewed in Baggio and Junqué (2019). Encouragingly, the frontostriatal circuit is known to be a site of significant neural plasticity, with both long-term potentiation (LTP) and long-term depression (LTD) observed at glutamatergic synapses on the dendrites of medium spiny neurons of the striatum (Di Filippo et al., 2009). This plasticity is thought to be a key driver of multiple aspects of learning and memory, including reinforcement learning, and is highly dependent on behavioral state (Stoetzner et al., 2010). Thus, designing cognitive tasks for use in CT that specifically target this circuit may allow for the induction of neural plasticity, improving functional connectivity and, subsequently, cognitive function. Similarly, changes within the hippocampus and connected regions have been shown to underlie memory impairments observed in PD (Baggio and Junqué, 2019). As these are also sites of significant neural plasticity following CT (Belleville and Bherer, 2012; Valkanova et al., 2014), tasks that target these circuits may similarly be able to lead to improvement in cognitive function for individuals with PD.

To date, several studies have investigated the benefits of CT for cognitive function in PD (outlined in Table 1). A recent Cochrane review evaluating the effectiveness of cognitive training for PD-MCI and PD-D identified 7 studies fitting their criteria, culminating in a total of 225 participants with variable intervention lengths (Orgeta et al., 2020). The review found “*no difference between people who received CT and people in the control groups in global cognition shortly after treatment ended and no convincing evidence of benefit in specific cognitive skills and no benefit shown in ADL or QoL*” (Orgeta et al., 2020). Whilst comprehensive, the strict criteria limiting assessment to randomized-control trials (RCT) meant several notable studies over the last decade were excluded from evaluation. Furthermore, inclusion was restricted to studies assessing function exclusively in PD-MCI and PD-D and did not assess potential benefits in individuals with PD without cognitive impairment, a population that may arguably derive the most benefit from a CT intervention. Finally, studies utilizing integrative multi-component approaches were also excluded. Whilst the results of these studies are difficult to compare to standard CT alone, they are still important to consider, as they represent intervention strategies that have potentially integrated and expanded upon current paradigms in order to optimize delivery. Given these limitations, a comprehensive review of the literature is needed to fully assess the benefits, and future potential, of CT in PD. Additionally, given differences in diagnosis, methodology and outcome measures between studies, recommendations for more uniform study design criteria are also required.

**TABLE 1:** Study methodology details of Cognitive training in Parkinson’s disease.

Source	Sample size	Method of Administration	CT intervention(s)	Duration	Cognitive outcome measure(s)	Results
Pen and Paper CT						
Mohlman et al. (2011)	**16 PD -MCI/D** **patients** Mean age: 62.71, S.D.: 7.32; 10M, 6F)	Worksheets + Audio CD’s	Attention Process Training APT-II intervention aimed to train sustained, selective, alternating and divided attention	90 minute sessions 1x/week for 4 weeks	*Executive Function/Attention* Stroop Color Word Test Controlled Oral Word Association Test (COWAT) Digit Span Forward TMT-B	Patients improved on all 4 cognitive tests, and average ratings of progress were positively correlated with magnitude of change on these measures.

Pena et al. (2014)	**44 PD patients total** *CT group* = 22 (Mean age: 67.6 (65.25-69.84); 9F, 13M) *Control group* = 22 (Mean age: 68.1 (64.93-71.32); 8F, 14M)	Pen and Paper REHACOP program for attention, memory, language, executive function, social cognition and processing speed	REHACOP Delivered in a group setting Control group performed group occupational activities, such as drawing, reading the news, etc.	60 minute sessions: 3x/week for 3 months *Attention unit*: 4 weeks *Memory unit*: 3 weeks *Language unit*: 3 weeks *Executive function unit*: 2 weeks *Social cognition unit*: 1 week	*Processing Speed:*TMT-A and Salthouse Letter Comparison Test *Verbal learning and memory:*Hopkins Verbal Learning Test (learning and long-term recall) *Visual learning and memory:*Brief Visual Memory Test (learning and long-term recall) *Executive function:*Stroop test (word-color and interference) *Theory of mind:*Happ test *Functional disability:*World Health Organization Disability Assessment Schedule II (WHO-DAS II), short version *Depression*: Global Depression Scale (GDS)	Bootstrapped analysis of variance showed significant differences in mean change scores in processing speed, visual memory, theory of mind and functional disability. Neither verbal learning and memory or executive function showed any difference between groups.

Díez-Cirarda et al. (2018)	**15 PD Participants**	Pen and Paper REHACOP	Integrative group-based cognitive intervention (REHACOP) Attention (sustained, selective, alternate, divided) 4 weeks Memory (verbal, visual learning, recall, recognition) 3 weeks Language (verbal fluency, synonyms/antonyms) 3 weeks Executive Function (cognitive planning, verbal reasoning) 2 weeks Social Cognition (moral dilemmas, TOMS) 1 week	1 hour sessions: 3x week for 13 weeks *Baseline* – T0 *Post-treatment* – T1 *Follow-up* (18 months) – T2	Outcome Measures T1 weighted MRI *Motor* UPDRS III *Processing Speed* Trail Making Test-A Salthouse Letter Comparison Test *Verbal Memory* Hopkins verbal learning test *Visual Memory* Brief Visual memory test (learning and recall) *Executive Function* Stroop Test Theory of Mind (TOM) – Happe test *Apathy* Lille Apathy Rating Scale *Depression* Geriatric Depression Scale *Functional Disability* WHO DAS II	CT group demonstrated increased performance in VM, VIM, EF and ToM and decreased functional disability at follow-up compared to baseline testing. Increased performance in VIM and EF at follow up (T2) compared with post-testing (T1). No significant changes in PS, VM, ToM, Apathy, Depression or functional disability. PD patients showed significant deterioration in UPDRS III and trend towards progression in disease on Hoehn and Yayr scale Increased brain functional connectivity and maintenance at T2 compared to T1, however, significant gray matter reduction and alterations of white matter integrity were found at T2

**Computer-based CT**						

Sinforiani et al. (2004)	**20 PD +/-MCI patients** (8F, 12M) Mean age: 68.9, S.D.: 7.1	TNP Software	Stimulate cognitive functions (attention, abstract reasoning, visuospatial abilities)	60 minute sessions 6 weeks (2x/week; 12 sessions total)	*Cognition* MMSE *Visual/Spatial Memory* Corsi’s test *Attention* Stroop’s test and Wisconsin card sorting test (WCST) *Verbal Memory* Babcocks story *Verbal Fluency* Phonological word fluency (FAS) *Executive Function* Raven’s matrices and Digit Span	Patients performed significantly better compared to baseline on Babcocks story, Raven’s matrices and phonological word fluency. Effects were maintained at 6-months follow-up

Edwards et al. (2013)	**87 PD patients total** *CT group* = 44 (Mean age: 69.4, S.D.: 7.8; 16F, 28M) *Control group* = 43 (Men age: 68.2, S.D.: 8.4; 17F, 26M)	InSight Program 5 different programs designed to improve information processing in realistic visual contexts.	CT = Self-administered speed of processing training Control = no contact.	60 minute sessions: 20 hours over 3 months, with every other session on the Road Tour exercise only	The Cognitive Self-Report Questionnaire Useful field of view test (UFOV)	CT group experienced significantly greater improvements in speed of processing than the control group. No differences between groups were seen on the Cognitive Self-Report Questionnaire.

Petrelli et al. (2014)	**65 PD Patients** *NEUROvitalis (NV) group* = 22 (Mean age: 69.2, S.D. 4.9; 12F, 10M) *Mentally fit (MF) group* = 22 (Mean age: 68.8, S.D.: 6.7; 7F, 15M) *Waitlist Control (CG) group* = 21 (Mean age: 69.1, S.D.: 11.6; 9F, 12M)	NEUROvitalis program Trained attention, memory, executive function	NEUROvitalis CT group - Individual tasks, group tasks and group games. Each session focused on one specific cognitive domain and started with a psychoeducational module. Mentally fit:Cognitive domains were not focused on in individual sessions. Instead, individual and group tasks for training attention, memory and less specific functions (general language, creative thinking) were combined randomly over the course of the entire program. Group conversations were used in place of psychoeducational sessions.Waitlist control group received no contact.	90 minute sessions:2x/week for 6 weeks (12 sessions total)	*Attention:*Brief test of attention *Memory:*Verbal short-term (DemText, Memo), Verbal long-term (DemTect, Memo), Visual long-term (Complex figure recall) *Executive functions:*Working memory (DemTect: digit span reverse), verbal fluency (semantic: DemTect; phonemic: FAS) *Visuoconstruction*: Figure copy *Quality of Life:*PDQ-39 *Depression*: Beck Depression Inventory-II	NEUROvitalis group demonstrated statistically significant improvements in short term and working memory (assessed by word list learning and digit span reverse, respectively). The increase in working memory was significantly greater than that in the Mentally fit group.

Petrelli et al. (2015)	**47 PD Patients** *NV group* = 16 (Mean age: 69.4, S.D. 4.2; 8F, 8M) *MF group* = 17 (Mean age: 68.6, S.D. 7.3; 5F, 12M) *CG* = 14 (Mean age: 68.8, S.D. 9.2; 5F, 9M)	NEUROvitalis program Petrelli et al. (2014) follow-up	Participants received no further intervention.	1 year follow-up	*Overall cognitive function:* MMSE DemTect *Responder:*Combined score of percentage change from baseline to 1-year follow-up *Risk of developing MCI*	Both the NV or MF groups maintained their DemTect score at 1-year post-follow up. Individuals in the NV group also maintained their MMSE score. While only 21.4% of the CG were classified as responders, 41.2% of the MF group and 56% of the NV group were responders. Patients without MCI at baseline from the CG had a risk of 40.0% to develop MCI from baseline to 1 year follow-up, while patients who received either intervention) had a risk of only 18.2%.

Alloni et al. (2018)	**31 PD + MCI Participants** CoRe CT (17) = (12F;5M, Mean Age: 71.2;SD: 7) Control Intervention (14) (5F;9M, Mean age 69.5;SD 8)	CoRe System	Computer-based logical-executive task Find the category Find the Intruder Unscramble the Images Image and Sound Word Coupling Logical Sequences Logical analogies Find the Elements Functional Planning Placebo Control intervention	45 minutes: 3x week for 4 weeks (12 sessions total) Baseline – T0 Post-treatment – T1 Follow-up (6 months) – T2	*Global Cognition* MMSE MOCA *Executive Function* Raven’s Matrices 47 test (RM47) Weigel’s Color-For Sorting Test (WCFT) Frontal Assessment Battery (FAB) F-A-S Test *Attention* Attentive Matrices Trail Making Tests A and B Stroop Test *Verbal Memory* Verbal Span Digit Span Logical Memory Test (immediate and delayed recall) Rey’s 15 word test (immediate and delayed recall) Rey’s Complex figure with delayed recall - RCF-dr) Wechsler Memory Scale WMS *Spatial Memory* Corsi Block-Tapping Test Rey-Osterrieth complex Figure Test *Visuo-spatial ability* Rey-Osterrieth Complex Figure Copy Test	After intervention (T0-T1), CT group improved significantly on MOCA compared to control interventions. Compared to baseline, CT group improved in 12/21 assessments (MoCA, R 15-word test recall, Logical Memory, Raven’s Matrices, Weigl’s,FAB,TMTa,TMTb,Stroop Tests (both), FAS, RCF-dr). Control group only improved in RCF-dr. At follow up (T1-T2) CT and control groups displayed significant worsening on MOCA and FAS, however, only the control group experienced worsening in MMSE and Logical Memory delay recall. No significant differences were observed between groups Overall, (T0-T2), CT intervention demonstrated significant improvements in Rey’s 15 word test (immediate), Weigl’s sorting test, Stroop test (time interference) compared to baseline, whilst control group only showed improvement in Rey Complex Figured (delayed recall), whilst worsening in MoCA, Digit Span, Raven’s Matrices, FAB, TMT A and Stroop test error interference. Improvements in CT were significant compared to controls in MOCA, Corsi’s, Reys 15 word test (immediate and delayed recall), Weigl’s test, TMTa and Stroop test.

Folkerts et al. (2018)	**12** **PD+D Participants** NEUROvitalis CT (6) = (5M;1F, Mean Age 76.67; SD 5.58) Control Group (6) (5M;1F, Mean Age 76.5; SD 8.94) (Randomised crossover trial)	NEUROvitalis System (Modified)	CT – Modified NEUROvitalis (modified version) Targets executive function and visual spatial function Control treatment - Usual care (includes sports, music and arts) - went on to receive CT	60 Minutes: 2x weekly/ 8 weeks (16 sessions in total) Post assessment + 6 week follow up	*Global Cognition* Consortium to Establish a Registry for Alzheimer’s Disease (CERAD) *Verbal Fluency* Word Fluency Test *Attention* TMT *Activities of Daily Living* Barthel Index *Quality of Life* QUALIDEM Scale *Depression* GDS Cornell Scale for Depression in Dementia (CSDD) *Health Related Quality of Life* EQ-5D-5L *Neuropsychiatric Symptoms* Neuropsychiatric Inventory (NPI)	Group differences favoured CT, with a trend for improvement in overall CERAD score and NPI, although these did not reach statistical significance (p=0.067 and 0.075 respectively). Compared to baseline, CT group demonstrated a trend for improvement in CERAD and GDS, however, these also failed to reach statistical significance (p=0.06 and 0.07 respectively).

Cerasa et al. (2014)	**15 PD Patients** *CT group* = 8 (Mean age: 61.1, S.D.: 12.4) *Controlgroup* = 7 (Mean age: 58.3, S.D.: 9.6)	Rehacom Software Training Attention and Information Processing Tasks	Rehacom as in Cerasa et al. (2013) Control group performed a simple visuomotor coordination tapping task	60 minutes sessions: 2x/week for 6 weeks (12 sessions total)	*Spatial memory:* ROCFT *Verbal memory:* Selective reminding test (SRT) *Visuospatial processing:* Judgment Line Orientation Test *Verbal fluency:* Controlled Oral Word Association Test *Sustained attention and information processing:* Symbol digit modality test and PASAT *Executive functions:* Digit span forward/backward, Stroop word-color task and TMT A and B *Mood*: Beck II, STAI-/y *General Cognition*: MMSE *Quality of Life:* PDQ-39	CT group showed improved cognitive performance compared to the control group on a measure of attention (Symbol-digit modality test) and executive function (digit span forward). These improvements were associated with significantly increased intrinsic functional activity in the left dorsolateral prefrontal cortex within the left central executive resting state network (RSN) and in the left superior parietal lobule within the attention RSN.

Fellman et al. (2020)	**52 PD +/- MCI Patients** CT Group (2) = (17M;9F, Mean age 64.8; SD 6.2) Active Control Group (26) = (17M;9F, Mean age 66.5, SD 4.7) Healthy Control Group (54) = (41M;13F, Mean age 66; SD 4.1)	Home training using Working Memory Tasks including: *N-back training task* *Selective Updating of Sentences Training Task (SUST) Forward Simple Spain Training Test (FSST).*	Home based RCT PD CT group = Working memory (WM) training Active control group = received quiz training (general knowledge) Healthy Control group = no intervention	30 Minute sessions: 3 x 30 minute sessions p/w for 5 weeks (5 stages over 8 weeks (1-3, pre-testing, 3-7 testing, 7-8 post-testing)	*WM* N-back training task Selective Updating of Sentences Training Task (SUST) Forward Simple Spain Training Test (FSST). Working memory questionnaire *Task specific near transfer tasks* N-back with colors Selective updating of digits (SUD) Forward color span *Task general near transfer* Running memory task AWM task Minus 2 span task *Far transfer* *Verbal Memory* Sentence Recall and Word List Recall *Executive Function and Attention* Continuous performance task (CPT) and Stroop test BRIEF-A Depressive Symptoms GDS-30	PD patients WM function was well-preserved, with performance comparable to healthy controls. PD patients were, however, impaired in self-assessment of WM and executive function. Compared to active controls, the WM CT group showed significant improvement in 2/3 WM tasks and near-transfer improvements, however, this did not translate to improvements in far transfer domains such as verbal memory, executive function and attention or self-assessed measures of WM and executive function. There was, however, a decrease in depression scores associated with WM CT.

**Combination – Pen & Paper + Computer-based (or unspecified)**						

Nombela et al. (2011)	**20 patient’s total** 10 PD 5 E-CT/5-C-CT (mean age: 60.5 SD 3.45, 6F, 4 F) **10 Healthy Control** (mean age: 59.6 SD 4.47, 6F, 4M)	Not Specified	Experimental CT = Modified Stroop Test Control CT = Sudoku Participants with tremor, dyskinesia or substantial motor impairment were not considered	Control CT One Sudoku table every day at home for 6 months	Cognitive Screening UPDRS MMSE Montgomery Asberg Depression Rating Scale (MADRS) *Attention* Stroop Test	Experimental (Trained) PD patients showed significant improvement in Stroop Test Reaction time compared to CT control and HC. This corresponded with attenuated pattern of brain activation

París et al. (2011)	**28 PD patient’s total** *CT group* = 16 (Mean age: 64.8, S.D: 9.2; 7M, 9F) *Control group* = 12 (Mean age: 65.4, S.D: 9.6; 7M, 5F) 50% of participants in both groups met criteria for MCI.	Combination SmartBrain Software (Tarraga et al., 2006) Individualized program	Paper-based homework exercises, consisting of 20 cognitive exercises Control group received speech therapy.	45 minute sessions: 4 weeks (3x week; 12 sessions total) Homework exercise (1x week; 4 sessions total)	*Cognitive screening:* MMSE and Addenbrooke Cognitive Examination *Attention and working memory:* Digits subtest of WAIS-III; California Verbal Learning Test (CVLT), 1^*st*^ trial *Information processing speed:* Symbol-Digit modalities test; Trail-Making-test A; Stroop Word subtest *Verbal memory*: CVLT-II Short-Delay and Long-Delay Free Recall) andLogical Memory subtest (WMS-III) *Verbal fluency*: Phonemic-FAS; Semantic- Animals *Learning:* CVLT-II (List A Total) *Visual Memory and Visuoconstructive Ability:* Rey Osterrieth Complex Figure Test (ROCFT) *Visuospatial abilities:* RBANSLine orientation subtest *Executive function:* Tower of London (TOL); Trail-Making-Test B (TMT-B); Stroop Interference subtest *Quality of Life:* PDQ-39 *Cognitive difficulties in ADLs:* CDS	CT group significantly improved in tests of: *Attention and working memory* (WAIS-III Digit Span Forward) *Processing speed* (Stroop Word subtest) *Visual memory/visuoconstructive abilities* (ROFCT) *Visuospatial abilities* (RBANS Line Orientation subtest) *Verbal fluency* (Semantic-Animals) *Executive function* (TOL- total moves and total correct; TMT-B) There were no significant improvements in self-rated quality of life or ADLs. No significant effects on overall cognitive function MMSE/ACE

Costa et al. (2014)	**17 PD patients with MCI and 8 healthy controls total** *Healthy Controls* = 8 (Mean age: 67.2, S.D.: 6.2) *CT group* = 9 (Mean age: 66.1, S.D.: 7.1) *Placebo group* = 8 (Mean age: 70.9, S.D.: 4.8)	Combination Aiming to train shifting ability in prospective memory tasks	Patients alternately select between stimuli (e.g., letters, numbers and shapes) belonging to different semantic categories or with different visual/spatial features 4 modules of increasing difficulty, with each module consisting of 3 sessions Control participants= language exercises (dictation and reordering of sentences) that did not vary in difficult and respiratory exercises	45 minute sessions: 3x/week for 4 weeks (12 sessions in total)	*Prospective memory (PM) [after*McDaniel et al. (2004)*] and* *Verbal Fluency/Shifting Ability* Alternate Fluency and TMT	Significant improvement in the experimental group in accuracy on the PM procedure and performance on alternate, but not phonemic fluency compared to baseline and placebo group

Vlagsma et al. (2020)	**43 PD +/- MCI patients** PD CT group - ReSET (24) = (14M;10F, Mean age 60.21; SD 10.42) PD Control CT -CogniPlus (19) = (13M;15F, Mean age 62.58, SD 8.84) Healthy Control (90) = (42M;48F, Mean age 58.97; SD 6.41)	Not specified ReSET Neurorehab sessions - involving strategy training to improve executive function	PD CT group = Cog Rehab using strategy training ReSET PD Control = computerized repetitive practice training for attention with Cogniplus Healthy Controls = no intervention	60 Minute sessions: 1-2 x week/14 weeks (14 sessions in total)	*Activities of Daily Living* Role Resumption List (RRL) *Executive Function* Treatment Goal Attainment (TGA) Dysexecutive Questionnaire (DEX) Brock Adaptive Functioning Questionnaire (BAF-Q) *Attention* TMTA *Verbal Memory* RAVLT (immediate and delayed recall) *Caregiver Burden* Zarit Burden Interview (ZBI) Quality of Life PDQ-39	Immediately following treatment (T0-T1), both groups reported significant improvement in executive function (TGA and DEX), with improvements greater with ReSET than Cogniplus. No differences were observed between CT groups on ADL (RRL), Quality of Life (PDQ-39), Caregiver burden (ZBI) or BAF-Q. At follow-up (T0-T2), both treatment groups maintained improvements in executive function (TGA and DEX) compared to baseline, however, there was no significant difference between groups.

**Integrated/Multi-Modal CT**						

Reuter et al. (2012)	**240 PD patients with MCI total** (Mean age: 64, S.D: 4) *Group A*: CT = 71 (35F, 36M) *Group B*: CT and transfer training = 75 (36F, 39M) *Group C*: CT, transfer training and motor training = 76 (36F, 40M)	Computer-based (Individually Tailored) Integrated with transfer training Targeting executive function and memory	1. CT Individually tailored based on scores on baseline tests. Set of tasks requiring executive and memory function 2. Transfer training 90 minute sessions: Goal: to manage daily life better and become more self-confident Composed according to baseline results and patient preferences Example tasks: go to grocery store, prepare a meal, pay a bill, look after a vegetable patch, etc. 3. Motor training: 60 minute sessions Goal: to improve coordination, strength, speed, perception and orientation. Composed according to individual capabilities and needs Example tasks: perform motor sequences, dual task performance, find items, remember hidden items, obstacle course completion with changing rules, mental imagery, aerobic training, etc. Caregivers received a 5-module educational training program.	60 minute sessions: 4 weeks in rehab center: 1. CT = 4x/week, at least 14 sessions in total 2. Transfer training = 3x/week, at least 10 sessions in total 3. Motor training: Minimum 10, maximum 12 sessions Followed by 6 months at home: 1. CT = 3 x 45 minute sessions per week (All groups) 2. Transfer training = 2x per week (Groups B and C) 3. Motor training = 2x per week (Group C)	*Primary outcome measure:* Alzheimer’s assessment scale cognition (ADAS-COG) *Secondary outcome measure:* Scale for Outcomes in Parkinson’s disease Cognition (SCOPA-COG) Information processing speed: Paced auditory serial addition test (PASAT) *Executive function:* BADS	All groups improved significantly on both the primary outcome measure (ADAS-COG), and the secondary outcome measure (SCOPA-COG), with Group C having the most benefit. At 6-mo follow-up, 50% of Group A, 31% of Group B and 28% of Group performed worse on the ADAS-COG compared to performance at discharge. Further improvement was observed in 21% patients of Group A, 37% patients of Group B, and 50% patients of Group C. At 6-mo follow-up, 70% of Group A, 80% of Group B, and 94% of Group C maintained their discharge-level performance on the SCOPA-COG. On the BADS-subscales, all groups showed improvement at discharge, with Group C showing the most improvement. At 6-mo post-completion, Groups A and B had lost most of their improvement, while Group C largely maintained their scores. On the PASAT, group A did not improve, while both Group B and Group C showed improvement, with Group C benefitting the most from training.

Naismith et al. (2013)	**50 PD patients total** *CT group* = 35 (Mean age: 68.5, S.D.: 7.1; 26F, 35M) *Waitlist control group* = 15 (Mean age: 64.9, S.D.: 6.5; 10F, 15M)	Computer-Based (Individually Tailored) Neuropsychological Education Approach to Remediation (NEAR) Program Integrated +/- Psychoeducation	1 hour: Psychoeducation, modified for PD [see Naismith et al. (2011)] 1 hour: CT using NEAR. individualized computer-based training program based upon their neuropsychological test results. COGPAK	2 hour group sessions 2x/week for 7 weeks (14 sessions in total)	*Primary outcome measure:* Logical Memory subtest of the Wechsler Memory Scale-III (immediate recall and memory retention) *Secondary outcome measure:* psychomotor speed and mental flexibility (TMT-A and TMT-B) and verbal fluency (COWAT) *Knowledge:* 20-item MCQ test based on psychoeducation sessions	CT was associated with improvements in learning and memory, as measured by the Logical memory test. There were no differences in secondary outcome measures or knowledge between the groups.

Biundo et al. (2015)	**24 PD patients total (16 completed follow-up at 16 weeks)** *CT + real tDCS* = 12 (7 completed) (Mean age: 69.1, S.D: 7.6; 1F, 6M) *CT + sham tDCS* = 12 (9 completed) (Mean age: 72.3, S.D: 4.1; 1F, 8M)	Computer-based RehaCom software (includes Adapted specialized keyboard) Integrated with tDCS	RehaCom Non-invasive tDCS of the left dorsolateral prefrontal cortex (direct current = 2mA, 20min/session)	30 minute sessions: 4x/week for 4 weeks	Repeatable Battery Assessment of Neuropsychological Status (RBANS) *Cognition*: MMSE *Executive Function/Attention*: Digit Span/Written Coding *Memory*: Immediate memory Index, Story learning test (delayed) *Visuospatial Function*: RBANS VS index *Language*: RBANS Language Index *Quality of Life*: PDQ8 *Depression*: BDI-II *Anxiety*: STAI-Y	Immediately following the intervention, there was a significant decrement in performance in the real tDCS group compared to the sham t-DCS group in attention/executive skills (Written coding test). At the follow-up in Week 16, there was a trend for better performance in the real t-DCS group in the story learning test and the immediate memory test. Reported decline in executive skills and improved attention and memory

Lawrence et al. (2018)	**42 PD+MCI Participants** Standard CT (5) = (3M;2F, Mean Age 68.14; SD 8.69) Tailored CT (6) = (4M;2F, Mean Age 65.57; SD 5.2) tDCS (7) = (5M; 2F, Mean Age 72; SD 6.45) Standard CT + tDCS (7) = (5M; 2F, Mean Age 63.57; SD 15.68) Tailored CT + tDCS (7) = (5M; 2F, Mean Age 67.43; SD 6.37) Control (6) = (4M; 2F, Mean Age 72.29; SD 6.21)	Computer-based Smartbrain Pro Integrated with tDCS	Smartbrain Pro CT Target’s attention, working memory, psychomotor speed, executive function, visuospatial ability Implemented as ‘standard’ and ‘tailored’ based on baseline testing Control group received no intervention	45 Minutes: CT= 3x weekly/4 weeks tDCS = 20min sessions, 1Xweek/4 weeks	*Global Cognition* MMSE Parkinson’s Disease Cognitive Rating Scale *Executive Function* Stockings of Cambridge (SOC) test Controlled Oral Word Association Test (COWAT) *Attention* Letter number sequencing test (LNS) Stroop Task *Verbal Memory* Hopkins Verbal Learning Test-Revised Paragraph Recall Test *Language* Boston Naming Test Similarities Test *Visuo-spatial ability* JLO test Hooper Visual Organisation Test (HVOT) *Activities of Daily Living* UPDRS Part II *Quality of Life* PDQ-39	Compared to baseline, participants who underwent standard CT improved in depression and ADL post-intervention, however, this was not maintained at follow-up. Overall, follow-up, participants improved in Verbal Memory and Visuo-spatial ability. Participants who underwent tailored CT improved in depression and this was maintained at follow-up. Overall, at follow-up the tailored CT group improved in Attention and maintained improvements in depression. The combination of standard CT + tDCS saw improvements in executive function, language and ADL beyond those observed with just tDCS post-intervention, however, only improvements in executive function were maintained at follow-up. The combination of tailored CT + tDCS saw improvements in executive function and language beyond those observed with just tDCS. At follow-up, improvements were observed in executive function, attention, verbal memory and language. Overall, the control group did not improve in any outcomes measured.

Bernini et al. (2019)	**41 PD+MCI** **Patients** *G1 – Physical rehab + CT with CoRE (17)* = *(7M;16F, Mean age 71.18; SD 7.04)* *G2 – Physical Rehab control group = 18 (11M;7F, mean age 69.33; SD 7.72)*	Computer-based CoRe System Integrated with physical rehabilitation	CT group = Computer-based logical-executive task (CoRe) Find the category Find the Intruder Unscramble the Images Image and Sound Word Coupling Logical Sequences Logical analogies Find the Elements Functional Planning Control group = standard physical rehabilitation involving warm-up, active and passive exercises to improve joints’ range of motion, stretching of abdomen, strengthening, postural, balance and control exercises	45 minutes: 3x week/4 weeks (12 sessions total) 6-month follow up Baseline – T0 Post-intervention – T1 6-month follow up – T2	*Primary Outcome - Global Cognitive Function* MMSE MoCA *Memory* Verbal Span(Verbal Span, Digit Span) Spatial Span (Corsi’s block-tapping test (CBTT) Verbal Long-term memory (Logical Memory Test immediate and delayed recall (Rey’s 15-word test immediate and delayed recall Spatial long-term memory (Rey complex figure delayed recall – RCF-dr) *Logical-executive functions* Non-verbal reasoning (Raven’s Matrices 1947 – RM47) Categorical abstract reasoning (Weigl’s sorting test) Frontal functionality (Frontal Assessment Battery (FAB) Semantic Fluency (animals, fruits, car brans) Phonological fluency (FAS) *Attention* Visual selective attention (Attentive Matrices) Simple speed processing and complex attention (Trail Making Test part A and B – TMT) Selective Attention/susceptibility to interference (Stroop test) *Visuospatial abilities* Rey complex figure copy 9RCF-copy) *Functional Status* Activities of Daily Living (ADL) + Instrumental (IADL) *Mood* Beck Depression Inventory *QOL* PDQ-8	After intervention, compared to baseline (T0-T1) G1 showed significant improvements in MoCA, Verbal LTM (Reys 15 + LMT-IDR), Categorical abstract reasoning and Phonological Fluency. G2 showed no significant changes. Compared to controls, G1 performed better than G2 on MoCA, Verbal LTM, categorical abstract reasoning, simple speed processing and complex attention(A), non-verbal reasoning and selective attention/susceptibility. Both groups showed motor improvements. At 6-month follow up (T1-T2), G1 showing significant worsening on MoCA and phonological fluency G2 showed worsening on MoCA and FAS but also MMSE and frontal functionality. Accordingly, no significant improvements were maintained at 6-month follow-up. Overall (T0-T2), compared to baseline, G1 showed improvements in MoCA, delayed and immediate recall and Weigl’s test for executive function, whilst control groups significantly deteriorated over-time in MMSE, MoCA and FAB. Compared to controls, 6-months post intervention CT appeared to significantly improve in global cognition, verbal LTM, executive function and attention. No significant changes in mood or QOL at were observed.

**Adaptive/Assistive Technology**						

Pompeu et al. (2012)	**32 PD patients total** CT group = 16 (Mean age: 68.6, S.D.: 8.0) Control group = 16 (Mean age: 66.2, S.D.: 8.3)	Computer-based Wii Fit + Global Exercises (Adaptive technology) Cognitive demands of the games included attention, working memory and performance management.	CT Group= 30 minutes of global exercises 30 minutes of playing 10 Wii Fit Games (5 per session, 2 trials per game) The control group received balance exercise therapy with exercises requiring the same movements and time required by each game.	60 minute sessions: 2x/week for 7 weeks (14 sessions total)	*Primary outcome:* independent performance of activities of daily living (UPDRS-II) Montreal Cognitive Assessment (MOCA)	Both groups demonstrated improvements in UPDRS-II and cognitive function. There were no statistically significant differences between the two groups.

Cerasa et al. (2014)	**15 PD patients total** *CT group* = 8 (Mean age: 61.1, S.D.: 12.4) *Controlgroup* = 7 (Mean age: 58.3, S.D.: 9.6)	Computer-based Rehacom Software (Adaptive keyboard) Training attention and information processing tasks	Rehacom as in Cerasa et al. (2013) Control group performed a simple visuomotor coordination tapping task	60 minutes sessions: 2x/week for 6 weeks (12 sessions total)	*Spatial memory:*ROCFT *Verbal memory:*Selective reminding test (SRT) *Visuospatial processing:*Judgment Line Orientation Test *Verbal fluency:*Controlled Oral Word Association Test *Sustained attention and information processing:*Symbol digit modality test and PASAT *Executive functions:* Digit span forward/backward, Stroop word-color task and Trail Making Test (TMT) A and B *Mood*: Beck II, STAI-Y *Quality of Life*: PDQ39 *General Cognition*: MMSE	CT group showed improved cognitive performance compared to the control group on a measure of attention (Symbol-digit modality test) and executive function (digit span forward). These improvements were associated with significantly increased intrinsic functional activity in the left dorsolateral prefrontal cortex within the left central executive resting state network (RSN) and in the left superior parietal lobule within the attention RSN.

Zimmermann et al. (2014)	**39 PD Patients** CT (CogniPlus) = 19 (Mean age, 69.9, S.D.: 6.3; 68% male) Control = 20 (Mean age: 66.3, S.D.: 66.3; 60% male)	Computer-based CogniPlus Software + Wii (Adaptive technology)	CogniPlus = 4 modules in a fixed order for 10 min/module: FOCUS (attention) NBACK (working memory) PLAND (planning and action) HIBIT (response inhibition) Control – Game Console = 4 sports games from Wii Sports Resort: Table Tennis, Swordplay, Archery, and Air Sports Both adapt difficulty to performance	40 Minute sessions: 3x/week for 4 weeks (12 sessions total)	*Attention and Working Memory:*Tests of Attentional Performance (alertness and working memory) *Executive function:*Trail Making test (B/A) *Visuoconstruction:*Block-Design test *Episodic memory:*California Verbal Learning test	Following intervention, individuals in the Wii group scored more highly for tests of attention than the CogniPlus group. There were also trends towards improvement in the Wii group in visuoconstruction and episodic memory.

Maggio et al. (2018)	**20 patients with PD +MCI** Experimental CT group (10) = (6M; 4F, Mean Age 69.9; SD 6.3) Control Group (10) = (4M; 6F, Mean Age 68.9; SD 8.2)	Computer-based BTS-Nirvana Virtual Reality (Adaptive technology)	CT Group = Semi-immersive Virtual-reality training with BTS-Nirvana Control Group = traditional CT with face-face interaction and paper-and-pencil activities	60 minutes: 3 x week/8 weeks (24 sessions total)	*Global Cognition* MMSE Addenbrooke Cognitive Examination-Revised (ACE-R) for detecting mild CI in attention, orientation, visual-spatial cognition, language and fluency and memory *Executive Function* Weigl test Frontal Assessment Battery (FAB) *Anxiety* Hamilton Rating Scale – Anxiety (HRS-A) *Depression* Geriatric Depression Scale (GDS)	Compared to baseline, VR CT group improved in measures of cognition, executive function, attention and orientation, memory, Verbal Fluency, Language and Visual-Spatial ability, with control group only improving in 1 measure of executive function, global cognition (ACE-R) and visuo-spatial ability (ACR-VS) Compared to controls, VR CT demonstrated improvements in global cognition, executive function and visuo-spatial abilities, with the control group demonstrating significant deterioration over-time. No significant differences in mood (GDS and HRS-a) were observed.

van de Weijer et al. (2020)	**41 PD +MCI Patients** CT (21) = (Mean age, 64.65; SD 7.4) Waitlist Control (20) = (Mean age, 64.01; SD 7.41)	Computer-based Parkin’Play (AquaSnap Program) Gamification (Adaptive technology)	Parkin’Play (AquaSnap) = an adaptive online CT gaming platform targeting attention, working memory episodic memory, psychomotor speed and executive function	30 Minute sessions, 3 x week for 12 weeks (recommended agenda – not fixed schedule) Voluntary gameplay weeks 12-24 Follow-up at 12 weeks	*Global Cognition (not reported individually)* Stroop Color and Word Test Category fluency and Letter Fluency Rey Auditory Verbal Learning Test Location Learning Test Judgement of Line Orientation Rey-Osterrieth Complex Figure Boston Naming Test MyCQ	Primary objective was to study feasibility of intervention, reporting strong accessibility and motivation for the program, with compliance and technical smoothness to be improved. Compared to waitlist controls, the CT group improved in global cognition after 24 weeks of training, however, this was not maintained at 12 weeks follow up

Here, we will expand on the latest findings, with search criteria incorporating studies utilizing both RCT and pre-post design, as well as single and multi-component interventions targeting cognitive function in PD. Exclusion criteria include studies where cognition was not the primary outcome measure and studies that specifically excluded cognitive impairments. Due to the nature of motor impairments in PD, particular attention will be paid to the method of administration, including assessment of traditional, computerized and multi-component interventions, as well as a look toward the potential future of adaptive and assistive technology. Specifically, recommendations for the use of CT in PD will be proposed as a guide for the design of future studies.

## Consideration 1: Method of Delivery

### Pen and Paper-Based CT

Due to the heterogenous and debilitating nature of motor impairments in PD, method of CT administration is an important factor to consider when designing and evaluating the efficacy of any cognitive rehabilitation program and, as such, addressing these considerations is particularly pertinent. Of the studies considered in this review, 2 specified the exclusive use of “pen and paper” for the administration of CT. An active control group trial conducted by Pena and colleagues in 2014 utilized a structured program of paper-pencil tasks in the form of a previously validated exercise book called REHACOP (Pena et al., 2014; Sanchez et al., 2014). Made up of 300 tasks administered by psychologists in a group setting, REHACOP was originally designed for schizophrenia and adapted to elderly populations to train attention, memory, processing speed, language, executive functioning, social cognition and functional ADL. Tasks from REHACOP were administered for 13 weeks in 60-min group training sessions 3×/week. Improvements were reported in processing speed, visual memory and theory of mind, and also appeared to generalize to everyday tasks, with significant improvement on a self-administered measure of functional disability. No improvements were noted in either measures of executive function or verbal learning/memory (Pena et al., 2014).

More recently, a 2018 follow-up study investigated the long-term effects of REHACOP at 18 months post-intervention, finding improved performance in verbal memory, visual memory and decreased functional disability were maintained at follow-up, in addition to the appearance of previously unseen improvements in executive function (Díez-Cirarda et al., 2018). Interestingly, these improvements were observed despite significant gray matter volume loss and reductions in frontal activity, as well as significant deterioration in Unified Parkinson’s Disease Rating Scale (UPDRS) III (motor score), indicating progression of disease. It is important to note, however, that these changes were compared to baseline, with no control group at follow-up for comparison. The initial 2014 study reported a 4.2% attrition rate, with positive comments collected in an unpublished focus group, indicating satisfaction with the program. Such program enjoyment is an important factor to consider in assessing the feasibility of such programs, as also highlighted by another pen-and-paper based study in this area (Mohlman et al., 2011).

Similar to the intervention using REHACOP, Mohlman et al. (2011) also drew upon the adaptation of a previously developed program, administering CT using an adapted version of the Attention Process Training II (APT-II), which targets attention (Mohlman et al., 2011). In particular, they investigated the feasibility and acceptance of the regime, which utilized audio CDs and written worksheets to administer and evaluate the program in both a clinic-based and home-based setting. In addition to improvements in executive function, attention and verbal fluency, researchers determined a high degree of acceptance and successful engagement in the program. In particular, self-rating of progress was positively related to post-training improvement. The study, however, excluded participants with cognitive impairment, defined by a score of <24 score on the MMSE and degree of motor impairment was not taken into consideration. This is particularly pertinent, as the study reported correlation of effort with MMSE scores, indicating those with better cognitive ability are more motivated to engage in the program. Subsequently, by excluding participants with cognitive impairment, this may be biasing the sample toward a population who are already highly motivated. Furthermore, the study reported a 14% attrition rate, with data from these participants not included in assessment of feasibility, resulting in a potentially positive skew of attitudes toward the program. Irrespective of this, these findings highlight the importance of a patient’s confidence in CT programs, with perceived progress an important predictor of motivation and subsequent success, a factor that should be taken into consideration when assessing the efficacy of different CT intervention strategies.

### Computer-Based CT

In terms of studies utilizing a solely computer-based regime, Sinforiani and colleagues conducted the initial pilot study of CT in PD in 2004 (Sinforiani et al., 2004): 20 PD patients with MCI underwent 12 × 1-h sessions of computer-based CT (TNP software) over 6 weeks. This regime delivered individualized exercises targeting attention, abstract reasoning and visuospatial function. At the end of the 12 sessions, participants performed significantly better on neuropsychological tests for verbal fluency, verbal memory and executive function, maintaining performance at a 6-month follow-up (Sinforiani et al., 2004). However, no improvements were observed on measures of global cognition or attention. Whilst participants reported increased self-confidence, the CT program was also combined with a motor rehabilitation regime, which may have influenced these results; additionally, there was no control group. Positively, researchers reported no attrition and commented on the employment of a mouse to ameliorate the need for fine motor activity; however, this was not directly assessed or compared. Together, these limitations make it difficult to assess the full extent of these benefits and to attribute them directly to employment of a computer-based CT program.

A number of studies have since implemented various CT programs utilizing cognitive rehabilitation software previously validated in other neurological disorders, such as dementia and stroke, including Cognitive Rehabilitation System (CoRe) (Alloni et al., 2018; Bernini et al., 2019), NEUROvitalis (Petrelli et al., 2014, 2015), Cogniplus (Zimmermann et al., 2014; Vlagsma et al., 2020) and Strategic Executive Training (ReSET) (Vlagsma et al., 2020).

The CoRE system computer-based CT program specifically targets executive function through a battery of 8 activities. A study by Alloni et al. (2018) assessed the efficacy in a cohort of PD patients with mild executive and/or cognitive impairment. Results were promising, revealing significant improvements in 12 out of the 21 assessments, including verbal and spatial memory, executive function, attention and global cognition, following intervention (Alloni et al., 2018). Unfortunately, a study assessing long-term benefits at 6-month follow-up noted improvements in global cognition and attention were no longer as pronounced, with significant worsening compared to immediate post-test; however, overall improvements were still maintained in measures of executive function and attention when compared to baseline. Furthermore, it is interesting to note that the losses were not as extensive as those observed in the control group, indicating preservation of function which may otherwise deteriorate with disease progression. The CoRe system underwent a usability assessment in healthy volunteers, demonstrating an overall positive score. Subsequently, this was repeated in a small cohort of PD patients (*n* = 6), where it was reported subjects were more entertained and involved in tasks including visual-stimuli, which led to the adaptation of exercises to include 3D graphics to allow for more complex interaction and improve engagement (Alloni et al., 2014).

Another CT program validated in a comparable population is NEUROvitalis. Originally developed for training in older populations with mild mental impairments, a NEUROvitalis “structured” program was adapted by Petrelli et al. (2014) to specifically target attention, memory and executive function, domains preferentially affected in PD (Petrelli et al., 2014). This study compared the efficacy of this “structured” approach (NEUROvitalis + psychoeducation program) to an unstructured “Mentally Fit (MF)” program. 65 PD patients were randomized either to one of the NEUROvitalis or MF groups and underwent 12 × 90-min group sessions over 6 weeks, or to a control group, receiving no contact. Immediately following intervention, both the MF and NEUROvitalis CT group improved in short-term, and WM compared to controls, with an increase in WM significantly greater with NEUROvitalis than MF (Petrelli et al., 2014). Interestingly, a significant decrease in depression was only noted in the MF group, an effect attributed to the social interactions associated with the strategy. At one-year post-intervention, on an assessment of overall cognitive function, 56.3% of the NEUROvitalis group and 41.3% of the MF group retained or improved their cognitive performance, compared to only 21.4% of the control (Petrelli et al., 2015). Excitingly, in both intervention groups, the chance of developing MCI (18.2% each) was lower than that of the control group (40.0%) (Petrelli et al., 2015). These results indicate CT could help prevent conversion to MCI in PD; however, the benefits of a specifically structured program designed to target domains affected in PD did not appear to be superior to the non-domain specific unstructured intervention.

A 2018 study went on to assess the efficacy of the NEUROvitalis program in a cohort of PD-D patients using a modified version of the program adapted for patients with dementia living in a nursing home and designed to target executive and visuo-spatial function (Folkerts et al., 2018). Results are preliminary due to the small sample size (*n* = 12) and failure to reach statistical significance (*p* = 0.067, *r* = 0.43); however, the strong effect size indicated the intervention may have been beneficial for cognition, although this was not maintained at a 6-week follow-up. Unfortunately, specific cognitive domains were not probed individually. In terms of supplementary outcomes, PD-D participants demonstrated potential improvements in both depression and ADL compared to baseline (Folkerts et al., 2018). This is in contrast to the earlier study in PD-MCI participants, possibly due to a ceiling effect, with milder cognitive impairments associated with reduced depression and impairments of ADL (Petrelli et al., 2014). This suggests adapting CT programs for their specific population of interest, as was done for the PD-D cohort, may help to optimize improvement and improve transferability to real-life applications.

Finally, Cogniplus is another computerized CT program shown to improve both attention and executive function in patients with MCI and schizophrenia; however, to date, its assessment in PD has been limited to use as a control intervention (Zimmermann et al., 2014; Vlagsma et al., 2020). Interestingly, Cogniplus was used as a cognitive-specific control in a study assessing the efficacy of a non-cognitive-specific, physically demanding, interactive video game (Wii Sports), which proved as, if not more, effective than Cogniplus training (Zimmermann et al., 2014). On the other hand, in a recent 2020 study by Vlagsma and colleagues, Cogniplus was utilized as a non-specific control intervention (*n* = 16) for a domain-specific psychoeducational program (ReSET) (*n* = 24) aimed at improving executive function in a cohort of PD patients with executive dysfunction. Significant improvements were observed in measures of both executive functioning and everyday life in both groups following treatment, as well as at 3–5 month follow-up, indicating no significant treatment effects and demonstrating Cogniplus to be as effective as a specifically designed program in improving executive function (Vlagsma et al., 2020).

### Combination of Pen and Paper and Computer-Based CT

Several CT programs over the last decade have also utilized a combination of “pen and paper” and computer-based delivery. París et al. (2011) were the first to conduct a study of CT in PD in which a control group received a placebo intervention (París et al., 2011). In this study, over the course of 4 weeks, the experimental group (*n* = 16) received 12 × 45 min CT sessions using interactive multimedia software (SmartBrain tool), as well as weekly paper and pencil-based homework exercises and a weekly tutoring session. The control group (*n* = 12) received speed therapy. Following the intervention, the CT group significantly improved in several cognitive domains (see Table 2 for summary outcomes) (París et al., 2011). However, there were no significant improvements in ADL or self-rated QoL (París et al., 2011), suggesting that, while CT may be beneficial for improvements on specific neuropsychological tests, these skills may not generalize to improvements in everyday functioning. Similarly, in a study investigating the effects of CT specifically targeting prospective memory (PM), Costa et al. (2014) utilized a combination of pen and paper and computer-based tests, finding the intervention improved performance on measures of executive function; however, generalizability was not assessed across other domains. It is difficult to comment on the acceptability of combined programs in the PD population from these studies alone; however, their investigations suggest the need for future studies utilizing this combination of techniques.

**TABLE 2:** Summary of Cognitive training in Parkinson’s disease study outcomes.

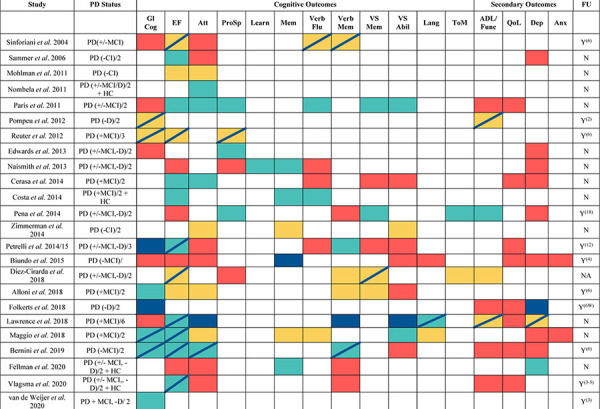

*Summary of CT study findings. Gl Cog = Global Cognition, EF = Executive Function, Att = Attention, Pro Sp = Processing Speed, Learn = Learning, Mem = Memory, Verb Flu = Verbal Fluency, Verb Mem = Verbal Memory, VS Abil = Visuospatial ability, Lang = Language, ToM = Theory of Mind, ADL =Activities of Daily Living/Function, QoL = Quality of Life, Dep = Depression, Anx = Anxiety, FU = Follow-up period^(*months*)^ unless stated otherwise. MCI = Mild Cognitive Impairment, CI = Cognitive Impairment, D = Dementia, HC = Healthy Controls. +/- indicates inclusion of participants with and without MCI/CI, - = exclusion of MCI/CI and/or D. + = specific inclusion of MCI and/or D. Categorisation of MCI/CI/D are based on varied criteria used by each study. Yellow = Improvements compared to baseline (if no control improvements), Green = Improvements compared to PD control group, Red = No Improvements, Navy Blue= Improvements/maintenance at follow-up. Navy Stripe. = Improvements/maintenance at follow-up. Several cognitive outcomes may have more than one measure, outcome is reported based on improvement in at least 1 test. In the “PD status” column, “/#” indicates the number of groups that participants were divided into.*

Overall, it is difficult to compare the efficacy of these different methods of administration interventions based on their cognitive outcomes alone, due to the significant variability in sample selection criteria, demographics, outcome measures and study duration. Furthermore, participants with dementia and those with severe motor impairments were excluded from the majority of studies above, making it difficult to interpret the role that method of administration may have on outcomes for these individuals. To date, there are also no studies which have directly assessed pen and paper vs computerized CT programs. However, there are several factors that may speak to the potential advantages of computer-based CT. In particular, with deterioration of handwriting (i.e., micrographia) considered a diagnostic sign of PD, pen and paper methodology carries a specific requirement of manual dexterity, which may be inherently difficult for PD patients who are severely motor impaired, thus likely to affect participation and outcomes for the population (Thomas et al., 2017). Additionally, significant advancements in technology have enabled the use of a variety of different modalities, including touch-screen, mobile-adapted, virtual reality and even interactive gaming. In addition to advantages for researchers in ease of delivery and analysis, the potential advantages of such technology-based interventions for patients include the ability to easily tailor interventions based on the individual’s needs, improved accessibility and interactivity, and the ability to modify, update and provide real-time feedback (Lampit et al., 2014). These may also assist in reducing fatigue, maintaining engagement and improving interaction with the program. This is significant, given evidence that enjoyment of the CT intervention may drive more beneficial outcomes (Mohlman et al., 2011).

In support of this, a recent systematic review of the use of CT for individuals with mild cognitive impairment concluded technology-based interventions demonstrated better effects than traditional “pen and paper” CT programs in improving function and QoL (Ge et al., 2018). Taken together, there is evidence to suggest CT may be a promising avenue for the non-pharmacological treatment of cognitive impairment in PD. In particular, computerized implementation represents a cost-effective and adaptable option and appears to now be the predominant approach.

## Consideration 2: Standard vs Tailored Delivery

The NEUROvitalis 2014 and 2018 studies assessed the efficacy of a CT program tailored to target domains dominant in cognitive dysfunction in PD and then further adapted these for specific sub-populations (PD-MCI and PD-D) (Petrelli et al., 2014; Folkerts et al., 2018). By tailoring CT delivery and refining investigations to appropriate outcome measures, this may improve the reliability of outcomes, which may otherwise be prone to ceiling effects in milder-PD populations.

Beyond this, a growing body of research has begun to investigate the potential to tailor CT programs not just to the specific population, but to the needs of the individual. The facilitation of tailored CT has been made much less resource intensive through the use of computer technology, which is able to assess impairments whilst simultaneously adapting the difficulty level and delivery of an otherwise-standard CT program, in order to target the deficits reflected by the individual. Studies discussed above (Sinforiani et al., 2004; Mohlman et al., 2011; París et al., 2011; Pena et al., 2014; Petrelli et al., 2014, 2015; Alloni et al., 2018; Díez-Cirarda et al., 2018) have all utilized a standard CT program. On the other hand, Naismith et al. (2013) used Neuropsychological Education Approach to Remediation (NEAR) to implement an individually tailored CT regime. NEAR was originally developed to address cognitive impairment in psychiatric disorders, targeting learning as its core domain, and includes an extensive software library of activities (Medalia and Freilich, 2008). Naismith et al. (2013) assessed the efficacy of NEAR in a cohort of PD patients with and without cognitive impairment (*n* = 35 intervention, *n* = 15 wait-list control). Delivery included 2 ′ 2-hour sessions per week over 7 weeks, paired with psychoeducation sessions, with waitlist control participants waiting 7-weeks before participation. Due to the online delivery platform, CT exercises were easily able to be tailored to the individual participant’s needs based on baseline testing (Naismith et al., 2013). Acceptability was high, with a low attrition rate of 4%, and significant improvements were noted in primary outcome measures of learning and memory; however, no changes were observed in psychomotor speed, executive function or depression, with no follow up to assess long-term benefits. This may again be due to the relatively mild cognitive impairment observed in the PD sample, representing a ceiling effect. Similarly, a study by Cerasa et al. (2014) also utilized a computer-based CT program (see Table 1 for details) targeting attention and information processing, which was tailored to individuals’ pretraining cognitive impairment(s). The intervention group demonstrated improvements in attention, which were also associated with increased functional magnetic resonance imaging (fMRI) activity in areas essential in executive function, providing additional support for a tailored approach (Cerasa et al., 2014).

Lawrence et al. (2018) were the first group to directly investigate the benefits of a standard vs tailored CT program in a PD population. PD participants (*n* = 7 per group) with diagnosed MCI received either computer-based training at home via Smartbrain Pro for 3 × 45 min per week for 4 weeks or a control (no intervention). CT was tailored based on baseline impairments, with standard training leading to improvements in memory, ADL and QoL, whereas the tailored intervention improved attention/WM and QoL (Lawrence et al., 2018). No improvements were observed with control intervention. This is the first study to report improvements in QoL with CT, with the former París et al. (2011) study also utilizing the Smartbrain tool finding no significant improvement, potentially due to implementation in a less severely impaired population (París et al., 2011). Whilst these results are preliminary given the small sample size and assessment comparison using only one program, they are cautiously indicative of the potential benefits of tailored CT. Additionally, it is worth noting that, although standard CT resulted in improvement in memory, this is a less-impaired domain in PD (Monastero et al., 2018). Improvements in attention/WM exhibited in the tailored program may in fact be more relevant for the PD and PD-MCI population, with a 2018 study identifying attention and executive function impairments affecting 39.5 and 28.5% of individuals, respectively, compared to a prevalence of 21.8% for memory (Monastero et al., 2018).

These results further support the use of computer-based technology, due to the ability to specifically tailor CT programs to the needs of the individual whilst prompting the need for future investigations into tailored CT programs. This may be particularly pertinent for further investigations of tailored CT efficacy on outcomes such as global cognition and depression, where improvements are often potentially overlooked due to the frequent exclusion of participants with moderate-severe CI and clinical depression, resulting in a ceiling effect. Taken together, tailored CT programs prompt a potential benefit in transferability due to their ability to target cognitive dysfunction in domains preferentially affected by the individual. This is perhaps most evident in the improvements observed in QoL, which have been otherwise been largely uninfluenced in standard CT programs.

## Consideration 3: Group-Based vs Home-Based Administration

Another variable which complicates the comparison of overall CT program efficacy are inconsistencies in administration. Many initial CT programs included administration by researchers or clinicians in a group-based clinical-setting; however, although computer-based CT may have improved adaptability and ease of use for PD patients, they have also resulted in an increased number of platforms available online and, as such, implemented in an unsupervised home-setting. This approach has been adopted, in part, as a less resource-intensive and cost effective alternative to lab-based CT (Fellman et al., 2020).

One such study conducted by Edwards et al. (2013) assessed the efficacy of a tailored CT program specifically targeting cognitive speed of processing training (SOPT) in a PD population using InSight software. Patients were randomized to receive 20 h of self-administered training over 3 months via InSight (*n* = 44), or a control (no-contact, *n* = 43). Results indicated greater performance in visual attention from control participants; however, both groups improved from baseline performance (Edwards et al., 2013). This, however, did not translate to previously reported improvements in secondary outcomes, such as improved cognition or depressive symptoms (Wolinsky et al., 2009). This may be due to the fact that only 69% of the intervention group completing the minimum required training hours. Importantly, a 15% attrition rate was reported in the study, which is higher than the ∼4% attrition rate reported in group-based CT studies in this population (Naismith et al., 2013; Pena et al., 2014) indicating that participants may be less motivated to complete the program when it is self-directed, as opposed to administered in a more guided and supportive manner.

A more recent study utilizing a home-based CT program was conducted by Fellman et al. (2020) to investigate the efficacy of an online CT program comprised of WM tasks aimed at improving WM and transfer to every-day. The groups received either WM training (intervention) or quiz training (control) in 3 × 30-min sessions/week for 5 weeks. Unfortunately, the population were relatively well-preserved with regards to WM, with performance comparable to healthy controls, making it difficult to assess benefits of the program. Nevertheless, compared to controls, the intervention group did demonstrate significant gains in two of three WM tasks (Fellman et al., 2020). Despite this, no transfer of benefit was seen in domains such as verbal episodic memory, executive function or attention. Furthermore, although a decrease in depression was observed, post-test self-assessment of WM function and executive function were unaffected, suggesting limited transfer effects to noticeable improvements in everyday life. With regards to acceptability of the program, the study observed a more favorable attrition rate of 8.6% compared to Edwards et al., with otherwise positive feedback and high adherence noted; however, the minimum number of sessions for inclusion were not specified. Overall, evidence for the efficacy of home-based delivery of CT in the PD population is currently limited, with potentially poorer translation to outcomes related to improved QoL and lower adherence to the CT protocol.

This is consistent with findings in cognitively healthy older adults, with a systematic review of computerized CT in this population concluding that group-based training was significantly more efficacious than home-based training, with home-based ineffective at improving cognitive performance. The authors attributed this to factors such as the ability to directly supervise participants in order to ensure adherence and compliance, to provide motivational support and encouragement and to problem solve IT-issues as they occur, as well as increased social interaction for participants (Lampit et al., 2014). This may be particularly advantageous for individuals with PD, who often experience disrupted social connectedness and social isolation (Soleimani et al., 2014), which is a major predictor of decreased health-related QoL in individuals with PD (Andreadou et al., 2011). Thus, group-based computerized CT may be indicated.

## Consideration 4: Standardization of Assessment Batteries

Another factor affecting whether or not a study may observe improvements with CT is dependent upon the assessment batteries used to evaluate outcomes. This may be best demonstrated by looking at differences in studies which have utilized an extensive assessment battery, with multiple tests used to assess a single domain. Alloni et al. (2018) and Bernini et al. (2019) are two recent examples of such studies. As discussed, Alloni and colleagues implemented a CT program and assessed outcomes using a total of 21 tests to evaluate cognition (2), verbal and spatial memory (8), executive function (5), attention (4), visuospatial ability (2) (Alloni et al., 2018). Compared to controls, CT resulted in 1/2 of cognition tests, 1/8 of verbal and spatial memory tests, 3/5 of executive function tests, 2/5 of attention tests and 0/2 of visuospatial function tests, with similar variability compared to follow up. A similar pattern was also observed in the Bernini, 2019 study (Bernini et al., 2019). Without such extensive post-intervention assessment, improvements in key domains may not have been identified.

The choice of which assessments are used to examine cognitive benefit may also be of critical importance. In support of this, improvements in cognitive function have been observed using the MoCA but not the MMSE (Alloni et al., 2018). Despite this, the MMSE is the most commonly used test to both screen for cognitive impairment in PD and to assess global cognition, as reflected in the majority of studies consulted for this review. This may represent a significant limitation, as the MMSE is considered to be less sensitive to changes and, as such, subtle improvements in cognition may have been missed. This highlights the need for further research to determine the most valid measures of outcome assessment for the relevant cognitive domains in the PD population, in order to inform the development of a standardized assessment battery. Such an effort would also allow for direct comparison of results between studies, which is currently quite complicated due to significant variability in study design and outcome assessment.

## Specific Recommendations for the Delivery of CT in the PD Population

Although the recent Cochrane review did not conclude a benefit of CT for cognitive impairment in PD (Orgeta et al., 2020), several reviews that take into consideration a larger subset of the literature have supported its potential (Leung et al., 2015; Díez-Cirarda et al., 2018). Our findings further support this; however, as discussed, there are several inconsistencies and limitations that limit the ability to directly compare the efficacy of CT programs. Despite this, there are a number of recommendations for future study design in order to improve the utility of CT programs for therapeutic use in PD. In summary, these include:

1. The use of computer-based technology to improve engagement, accessibility and CT delivery.
2. Tailoring of CT programs to suit the cognitive domains predominantly affected in the specific sub-population of PD (PD-MCI/PD-D), in addition to tailoring based on the specific cognitive impairment demonstrated by the individual.
3. Use of group-based (rather than home-based) training, in order to encourage compliance and social interaction.
4. Refinement and standardization of assessment batteries, including the use of non-cognitive batteries, such as QoL and ADL, in order to better assess real-world transferability.

## The Future of CT for PD

In addition to the recommendations above, in order to further improve the delivery and efficacy of CT programs, specific consideration should also be given to developing technologies that better adapt the CT platform to the unique needs and physical limitations of the PD population. One way this may be done is via multi-modal techniques, incorporating CT with interventions targeting the neural mechanisms that underlie cognitive function. Evidence in healthy aging supports this potential, with older participants who walked on a treadmill while playing a spatial navigation game demonstrating stability of hippocampal volume over a 4-month training period, whilst volumes in the control population deteriorated (Lövdén et al., 2010). Potential techniques identified that lend themselves to integration include transcranial Direct-Current Stimulation (tDCS) and exercise/aeorobic training.

### Multimodal Delivery: CT + Transcranial Direct-Current Stimulation

Non-invasive brain stimulation via transcranial Direct-Current Stimulation (tDCS) may prove an effective technique to pair with CT due to its potential to facilitate neuronal plasticity, amongst other potential mechanisms (Mohammadi, 2016). Initial studies have reported improved cognition following tDCS in PD, including improvements in WM (Boggio et al., 2006) and executive function (Pereira et al., 2013) with tDCS of the dorsolateral prefrontal cortex (DLPFC). In a comparable study by Doruk et al. (2014), results also appear to be maintained up to 1 month following intervention (Doruk et al., 2014). It is theorized tDCS of the prefrontal cortex may enhance declarative and long-term memory consolidation (Javadi et al., 2014). These studies were in cohorts of cognitively healthy PD patients; however, a study combining tDCS with physical rehabilitation (PR) in PD-MCI reported reduction of depressive symptoms, as well as improvements in motor ability (PR ± tDCS), cognition and verbal fluency (PR + tDCS only), which were all stable at 3-month follow-up (Manenti et al., 2016). Taken together, these results suggest a promising outlook for tDCS for improving cognitive outcomes in both cognitively healthy PD and PD-MCI patients; however, its efficacy in a PD-D cohort remains to be seen.

Due to these promising results, it is hypothesized that pairing tDCS with CT may synergistically boost the treatment effect of either intervention alone. The first study to do so paired a computer-based program incorporating attention and information processing tasks (RehaCom) with tDCS in a PD-MCI cohort with 30-min sessions 4 times per week for 4 weeks. Interestingly, initially, a significant decrement in performance on attention and executive tasks was observed with tDCS compared to sham; however, at 16-week follow-up, a strong trend toward improved memory and attention performance was observed with tDCS + CT compared to CT alone, although a decline in executive skills was reported (Biundo et al., 2015). Similarly, in a recent study by Lawrence and colleagues, while no difference between standard or tailored CT was observed, participants receiving both standard or tailored CT in combination with tDCS demonstrated the most substantial benefit overall, with improvements in executive function, attention, working memory and ADL (Lawrence et al., 2018). Taken together, results suggest pairing CT with non-invasive brain stimulation via tDCS, specifically of the left dorsolateral prefrontal cortex, may improve performance compared to CT alone; however, in order to determine the ideal tDCS parameters and specific CT technique for the most effective translation to therapeutic delivery, further investigations are required (Biundo et al., 2015).

### Multimodal Delivery: CT + Exercise

Exercise is a common non-pharmacological intervention for neurodegenerative diseases, particularly PD (Crotty and Schwarzschild, 2020). Aerobic training is believed to promote neural rearrangement and, as such, may complement and enhance the efficacy of cognitive rehabilitation programs. While the cellular mechanisms via which these neuroplastic effects occur are still unclear, they may involve enhanced neurogenesis/synaptogenesis (Valkanova et al., 2014) or increases in myelination (Song et al., 2005). Physical exercise leads to increased levels of neural growth factors, including BDNF), which is essential for facilitating neurogenesis, cell survival and SP (Gomez-Pinilla et al., 2008). Thus, physical exercise may promote neurogenesis/synaptogenesis and CT may promote the survival of these cells and synapses (Nuechterlein et al., 2016). In support of this, several recent studies have reported beneficial effects when combining CT with aerobic exercise in schizophrenia (Oertel-Knochel et al., 2014; Malchow et al., 2015; Nuechterlein et al., 2016).

In PD, a study by Reuter and colleagues in 2012 adopted an individually tailored multimodal cognitive rehabilitation program. The CT involved a set of well-established “pen and paper” neuropsychological batteries, such as the Behavioral Assessment of Dysexecutive Syndrome (BADS) and Raven’s Progressive Matrices, which target executive and memory functions, as well as computer-based exercises. This was paired with transfer training and transfer + psychomotor endurance training, in order to investigate the transferability of post-CT cognitive improvements into everyday life (Reuter et al., 2012). In a cohort of PD-MCI participants, immediately following treatment, all groups improved in measures of global cognition and specific domains, such as executive function. Additionally, participants who received CT paired with transfer + psychomotor endurance training showing the most significant improvements, persisting for up to 6-months (Reuter et al., 2012). They also reported less PD-specific impairments following intervention, indicating improved QOL. In terms of compliance, participants who received CT combined with psychomotor endurance training were also more likely to continue training at home (90%) compared to those only receiving CT (60%). Thus, a multimodal approach may be superior to “pen and paper” based activities based on both compliance and outcomes, indicating integration of CT with motor training may be a promising future direction.

Another study which explored CT in combination with physical rehabilitation was Bernini et al. (2019), who utilized the CoRe system previously discussed. The study combined CoRe with cardiovascular activities and exercises designed to improve range of motion, balance and postural control. In a cohort of PD-MCI participants, CT + physical rehabilitation resulted in improvements in global cognition and executive function compared to baseline, both immediately following intervention and at 6-month follow-up (Bernini et al., 2019). This improvement was also significant compared to controls receiving only physical rehabilitation. The inclusion of physical rehabilitation also led to an improvement in motor performance for both groups. Overall, individuals receiving CT + physical rehabilitation also showed less cognitive decline than those who received physical rehabilitation alone, who displayed a significant worsening of cognitive function over time, signifying a potential delay in cognitive disease progression. Taken together, studies in healthy aging, schizophrenia and PD provide support for the integration of physical rehabilitation with cognitive training to improve outcomes. This is particularly apt for PD patients, given the defining accompaniment of motor dysfunctions observed in the population. Physical rehabilitation and exercise in PD patients is already a well-established non-pharmaceutical intervention for the motor impairments of PD and studies incorporating the two only provide further support for its integration to potentially target cognitive impairments (Reuter et al., 2012; Bernini et al., 2019). Building from this, whilst a promising future direction, there may be a way to further improve efficacy in PD by incorporating the benefits of motor-training with the physical delivery of CT programs themselves through the use of technology specifically adapted for the PD population.

## The Use of Adaptive and Assistive Technology to Deliver CT in PD

To date, the vast majority of studies of CT in PD have typically used either a manual “pen and paper” approach or a computer-based approach with standard keyboard and mouse functionality. This may represent a substantial and under-addressed barrier for the successful implementation and assessment of CT in the population. In support of this, a 2010 survey found that nearly 80% of PC-users with PD have significant and severe difficulties using a computer due to their illness (Nes Begnum, 2010). In particular, muscle stiffness, inertia and tremor were frequent problems, resulting in significant-highly severe difficulties using a standard mouse (42%) and keyboard (27%). This represents a significant barrier to the current technical delivery of CT in PD, potentially altering the successful evaluation of outcomes, as well as prospective benefits. Consequently, not only should commercially available CT programs be adapted to address the cognitive dysfunctions specific to PD patients, but technical implementation should also be approached in light of the restrictions imposed by the often-debilitating motor impairments. To address this concern, this may involve the use of currently available technologies for adaptation, or the optimization of new assistive technologies to aid in delivery.

While still a critically under-researched area, a few studies have begun to look at CT delivery utilizing adapted hardware. For example, Cerasa et al. (2014) used a specialized keyboard designed for severe motor impairment, which incorporates large buttons for navigation and selection, in order to deliver the RehaCom software, a program targeting attention and information processing. Over six weeks, non-demented PD patients with attentional deficits underwent either RehaCom CT (*n* = 8) or completed a visuomotor coordination task 2×/week. Improvements were seen in the CT group on measures of attention, which were associated with significantly increased intrinsic functional activity in the left dorsolateral prefrontal cortex within the left central executive resting state network (RSN) and in the left superior parietal lobule within the attention RSN (Cerasa et al., 2014). These brain areas are essential for executive function, particularly WM. Thus, increased activation in these areas could represent a compensatory strategy, allowing for enhanced performance in these cognitive domains. Whilst results appear promising, it is difficult to determine if the specialized keyboard played a part in the improvements observed, beyond what would otherwise be seen with the RehaCom software alone. In fact, another study utilizing the RehaCom software to deliver CT in a PD cohort also reported improvements in both memory and attention, although these were less than those observed when paired with tDCS (Biundo et al., 2015). In order to fully assess potential benefits, a comparison of outcomes obtained with the adapted keyboard, compared to traditional mouse/keyboard delivery, is needed.

Another tool that may prove useful for addressing the barriers of CT in PD is the Nintendo Wii^TM^. The Nintendo Wii^TM^ has been proposed as a tool for balance training in the elderly and those with motor impairments (Pessoa et al., 2014). In addition to potential motor improvements, due to the complexity of tasks in already developed Wii^TM^ Fit compatible games, it has been postulated that the platform may improve integration of motor and cognitive abilities in order to improve ADLs (Pompeu et al., 2012). Subsequently, two studies have used Nintendo Wii^TM^ consoles to deliver CT in PD. In the first of these, the experimental group (*n* = 16) used a Nintendo Wii^TM^ for 1-h training sessions 2×/week for seven weeks, while the control group (*n* = 16) received balance exercise therapy (Pompeu et al., 2012). Following intervention, both the CT and the control group demonstrated improvements in cognitive function and ADL from baseline; however, there were no statistically significant differences between the two groups in terms of cognition, indicating comparable outcomes for both the Nintendo Wii^TM^ and balance exercise therapy (Pompeu et al., 2012). Conversely, in another study using the Wii^TM^ console, Zimmermann et al. (2014) randomized PD patients to either a computer program specifically designed to improve cognition (CogniPlus, *n* = 19) or a Nintendo Wii^TM^ game console (*n* = 20) (Zimmermann et al., 2014). Participants utilized their respective CT devices for 40 min, 3×/week for four weeks. Following intervention, the only statistically significant difference between the groups was that individuals in the Wii^TM^ group scored higher on tests of attention than the CogniPlus group (Zimmermann et al., 2014) indicating commercially available gaming consoles may be as effective as specifically designed computer interventions for attention; however, larger scale studies, long-term follow-up assessments, and comparisons with other CT programs are necessary in order to fully evaluate this.

Incorporating virtual reality (VR) technology into the delivery of CT is another up-and-coming area of research. Several studies have established the efficacy of VR training for the rehabilitation of motor function in PD; however, the effects on cognitive outcomes are not well studied (Mirelman et al., 2013; Cikajlo and Peterlin Potisk, 2019). BTS Nirvana is a VR system that delivers a 3-dimensional multisensory simulation that can be used for interactive training and that has been designed to specifically target executive function, attention and visuospatial skills (Maggio et al., 2018). Using this system, a cohort of PD-MCI patients participated in 60-min sessions 3×/week for 8 weeks, with the control group taking part in a traditional pen and paper CT program for the same amount of time. The authors noted a greater improvement in executive and visuospatial abilities in those using the BTS Nirvana system compared to controls, concluding VR may represent an innovative direction to improve cognitive outcomes for PD patients (Maggio et al., 2018). Despite this, given the small sample size and lack of long-term follow up, as well as the lack of supporting studies, it is difficult to draw any definitive conclusions, although this represents an exciting area for future research.

As established previously, compliance and acceptability are important factors when considering the effectiveness of CT programs. VR is a delivery method that may promote engagement due to its immersive nature; however, gamification may be another way in which enhanced interactivity could potentially improve confidence, engagement, and compliance. Whilst several computer-based CT programs incorporate elements of gameplay, such as real-time feedback, they do not necessarily include key features often attributed to traditional video games. Potential benefits of gamification include high-score and reward incentives, personalization, self-directed challenge, exploration and free-play (Nagle et al., 2015). These are particularly important for the PD population, where patients have a decreased reward sensitivity in an off-dopaminergic medication state, as well as increased apathy (Muhammed et al., 2016). Therefore, enhancement of the rewarding elements of game play may improve perceived self-efficacy, motivation and adherence, subsequently improving outcomes (Van De Weijer et al., 2019). Whilst this area of CT adaptation is in its infancy, one recent 2020 study investigated the efficacy of a gamified CT program called “Parkin’Play” in a cohort of PD-MCI patients (*n* = 21) against a no-intervention waitlist control (*n* = 20). The program consisted of an online CT game (called “AquaSnap”) that was required to be played by participants at home for 12 weeks *ad hoc*, followed by a supplementary voluntary phase (weeks 12–24). In terms of feasibility, the study demonstrated moderate compliance, with an average of 98.3%; however, the compliance rate reduced to 68.3% when sessions included were individually capped at 36. The authors also reported successful accessibility (100%), as well as successful motivation, with 87.5% reporting a positive acceptability score. After 24 weeks of training, the intervention group improved in global cognition compared to controls; however, this was not stable at 12 weeks follow-up (van de Weijer et al., 2020). Although preliminary, these results suggest a need to further investigate the value of gamification for the implementation of CT in PD. Furthermore, gamification may also be integrated with other multi-modal interventions, such as exercise or assistive technology, to further enhance its potential to target cognitive outcomes in PD patients.

While interpretation should be cautious given the small number of studies and participants and significant variations in methodologies, the literature appears to support the use of CT in PD. In a recent meta-analysis on the use of CT in PD, of seven studies included in the final analysis (*n* = 272 participants across all studies), the overall effect on cognitive function was small, but statistically significant, and there were a number of significant improvements in several specific cognitive domains, including WM, executive functions and processing speed (Leung et al., 2015). Global cognition, memory, visuospatial skills and attention, however, were not significantly improved (Leung et al., 2015). Additionally, there were no significant improvements in either Independent ADLs or QOL measures (Leung et al., 2015). This suggests that more targeted CT approaches for areas such as memory, visuospatial skills and attention may be needed, and that further focus needs to be placed on enhancing the transferability and length of benefit of CT.

In addition, future work should assess which individuals with PD are most likely to benefit from CT in PD. Previous work has suggested that individuals with specific motor subtypes of PD may be at increased risk of cognitive impairment. In support of this, individuals with the postural instability-gait difficulty motor subtype of PD are over-represented in PD-D and show a faster rate of cognitive decline compared to those with the tremor dominant motor subtype (Burn et al., 2006; Arie et al., 2017). This suggests that the early implementation of CT for individuals with balance and gait disturbance may be particularly likely to yield benefit. Similarly, the beneficial effects of CT may vary in a sex-specific manner. Previous work in rodents has shown that intermittent CT enhanced cognitive performance on a practiced T-maze task in aged rodents of both sexes, but these benefits only transferred to *novel* cognitive tasks in females (Talboom et al., 2014). Comparably, in a study looking at cognitive training effects in individuals with amnestic MCI, cognitive training benefits were larger for working memory and both immediate and delayed verbal episodic memory in females compared to males (Rahe et al., 2015). Enhanced benefit in females has also been reported in those with established dementia following cognitive stimulation therapy (Aguirre et al., 2013). However, these results should be interpreted with caution, as it may be due to the types of tasks used to assess cognitive benefit, with previous research showing that females perform better on tasks of verbal episodic memory, while males are more likely to excel on tests of visuospatial episodic memory (Beinhoff et al., 2008). In addition, given the paucity of research that has looked at sex-specific effects following CT, and the limitations imposed by small sample numbers in probing such effects in existing studies, future work will be needed to assess whether this same effect holds true in individuals with PD, as well as the brain basis of such an effect.

## Conclusion

A number of considerations have been put forward in this review regarding study design, with the overarching goal of identifying the most effective CT technique for clinical translation. Efficacy may potentially be enhanced through combination with other evidence-based non-pharmacological strategies, such as exercise and tDCS, which may further compound the alterations in neural mechanisms that underlie CT benefits. Additionally, given patients’ impairments in manual dexterity, which may significantly hamper ability to use standard equipment involved in CT, focus should be given to developing CT delivery equipment appropriate for use in this population. For example, adding a sensory feedback component to CT delivery equipment may also prove beneficial, as the coupling of hand position, sensory feedback and controlled hand movements with cognitive stimulation has been shown to heighten hand-brain connectivity in a variety of neurological conditions (Borstad et al., 2013). With refinement of delivery mechanism and standardization of study protocols, CT may lead to notable improvements in cognitive function, or even delay the onset of PD-MCI or PD-D, an outcome that would be particularly critical given the limitations of current pharmacological approaches to improve declines in cognitive performance in PD.

## Author Contributions

BG conducted the literature search and drafted the manuscript. DH supervised the project and revised the manuscript. LC-P independently confirmed the results of the literature search, supervised the project, and assisted with initial drafting and revised the manuscript. All authors contributed to the article and approved the submitted version.

## Conflict of Interest

The authors declare that the research was conducted in the absence of any commercial or financial relationships that could be construed as a potential conflict of interest.

## Publisher’s Note

All claims expressed in this article are solely those of the authors and do not necessarily represent those of their affiliated organizations, or those of the publisher, the editors and the reviewers. Any product that may be evaluated in this article, or claim that may be made by its manufacturer, is not guaranteed or endorsed by the publisher.

## References

[B1] Aarsland D. (2016). Cognitive impairment in Parkinson’s disease and dementia with Lewy bodies. *Parkinsonism Relat. Disord.* 22 Suppl. 1 S144–S148. 26411499 10.1016/j.parkreldis.2015.09.034

[B2] Aarsland D. Andersen K. Larsen J. P. Lolk A. Nielsen H. Kragh-Sørensen P. (2001). Risk of dementia in Parkinson’s disease a community-based, prospective study. *Neurology* 56 730–736. 10.1212/wnl.56.6.730 11274306

[B3] Aarsland D. Bronnick K. Williams-Gray C. Weintraub D. Marder K. Kulisevsky J. (2010). Mild cognitive impairment in Parkinson disease: a multicenter pooled analysis. *Neurology* 75 1062–1069. 10.1212/wnl.0b013e3181f39d0e 20855849 PMC2942065

[B4] Aguirre E. Hoare Z. Streater A. Spector A. Woods B. Hoe J. (2013). Cognitive stimulation therapy (CST) for people with dementia–who benefits most? *Int. J. Geriatr. Psychiatry* 28 284–290. 10.1002/gps.3823 22573599

[B5] Akbar U. Friedman J. H. (2015). Recognition and treatment of neuropsychiatric disturbances in Parkinson’s disease. *Expert Rev. Neurother.* 15 1053–1065. 26289491 10.1586/14737175.2015.1077703

[B6] Alloni A. Quaglini S. Panzarasa S. Sinforiani E. Bernini S. (2018). Evaluation of an ontology-based system for computerized cognitive rehabilitation. *Int. J. Med. Inform.* 115 64–72. 10.1016/j.ijmedinf.2018.04.005 29779721

[B7] Alloni A. Tost D. Panzarasa S. Zucchella C. Quaglini S. (2014). “Enhancing computerized cognitive rehabilitation with 3D solutions,” in *Proceedings of the 8th International Conference on Pervasive Computing Technologies for Healthcare*, (Brussels: Institute for Computer Sciences, Social-Informatics and Telecommunications Engineering).

[B8] Andreadou E. Anagnostouli M. Vasdekis V. Kararizou E. Rentzos M. Kontaxis T. (2011). The impact of comorbidity and other clinical and sociodemographic factors on health-related quality of life in Greek patients with Parkinson’s disease. *Aging Ment. Health* 15 913–921. 10.1080/13607863.2011.569477 21547746

[B9] Arie L. Herman T. Shema-Shiratzky S. Giladi N. Hausdorff J. M. (2017). Do cognition and other non-motor symptoms decline similarly among patients with Parkinson’s disease motor subtypes? Findings from a 5-year prospective study. *J. Neurol.* 264 2149–2157. 28879438 10.1007/s00415-017-8605-x

[B10] Baggio H. C. Junqué C. (2019). Functional MRI in Parkinson’s disease cognitive impairment. *Int. Rev. Neurobiol.* 144 29–58. 30638456 10.1016/bs.irn.2018.09.010

[B11] Beinhoff U. Tumani H. Brettschneider J. Bittner D. Riepe M. W. (2008). Gender-specificities in Alzheimer’s disease and mild cognitive impairment. *J. Neurol.* 255 117–122. 18202815 10.1007/s00415-008-0726-9

[B12] Belleville S. Bherer L. (2012). Biomarkers of cognitive training effects in aging. *Curr. Transl. Geriatr. Exp. Gerontol. Rep.* 1 104–110. 10.1007/s13670-012-0014-5 23864998 PMC3693427

[B13] Benito-Leon J. Louis E. D. Posada I. J. Sanchez-Ferro A. Trincado R. Villarejo A. (2011). Population-based case-control study of cognitive function in early Parkinson’s disease (NEDICES). *J. Neurol. Sci.* 310 176–182. 10.1016/j.jns.2011.06.054 21774946

[B14] Bernini S. Alloni A. Panzarasa S. Picascia M. Quaglini S. Tassorelli C. (2019). A computer-based cognitive training in mild cognitive impairment in Parkinson’s disease. *NeuroRehabilitation* 44 555–567. 10.3233/nre-192714 31256092

[B15] Biundo R. Weis L. Fiorenzato E. Antonini A. (2017). Cognitive rehabilitation in Parkinson’s disease: is it feasible? *Arch. Clin. Neuropsychol.* 32 840–860. 10.1093/arclin/acx092 28961738

[B16] Biundo R. Weis L. Fiorenzato E. Gentile G. Giglio M. Schifano R. (2015). Double-blind randomized trial of t-DCS versus sham in Parkinson patients with mild cognitive impairment receiving cognitive training. *Brain Stimul.* 8 1223–1225. 10.1016/j.brs.2015.07.043 26319357

[B17] Boggio P. S. Ferrucci R. Rigonatti S. P. Covre P. Nitsche M. Pascual-Leone A. (2006). Effects of transcranial direct current stimulation on working memory in patients with Parkinson’s disease. *J. Neurol. Sci.* 249 31–38. 16843494 10.1016/j.jns.2006.05.062

[B18] Bohnen N. I. Albin R. L. (2011). The cholinergic system and Parkinson disease. *Behav. Brain Res.* 221 564–573. 10.1016/j.bbr.2009.12.048 20060022 PMC2888997

[B19] Bohnen N. I. Kaufer D. I. Hendrickson R. Ivanco L. S. Lopresti B. J. Constantine G. M. (2006). Cognitive correlates of cortical cholinergic denervation in Parkinson’s disease and parkinsonian dementia. *J. Neurol.* 253 242–247. 10.1007/s00415-005-0971-0 16133720

[B20] Borstad A. L. Bird T. Choi S. Goodman L. Schmalbrock P. Nichols-Larsen D. S. (2013). Sensorimotor training and neural reorganization after stroke: a case series. *J. Neurol. Phys. Ther.* 37 27–36. 10.1097/npt.0b013e318283de0d 23399924 PMC3726277

[B21] Brusa L. Tiraboschi P. Koch G. Peppe A. Pierantozzi M. Ruggieri S. (2005). Pergolide effect on cognitive functions in early-mild Parkinson’s disease. *J. Neural Transm.* 112 231–237. 10.1007/s00702-004-0193-0 15365788

[B22] Burn D. J. Rowan E. N. Allan L. M. Molloy S. O’brien J. T. Mckeith I. G. (2006). Motor subtype and cognitive decline in Parkinson’s disease, Parkinson’s disease with dementia, and dementia with Lewy bodies. *J. Neurol. Neurosurg. Psychiatry* 77 585–589. 10.1136/jnnp.2005.081711 16614017 PMC2117449

[B23] Calleo J. Burrows C. Levin H. Marsh L. Lai E. York M. K. (2012). Cognitive rehabilitation for executive dysfunction in Parkinson’s disease: application and current directions. *Parkinsons Dis.* 2012:512892. 10.1155/2012/512892 PMC3216311 22135762

[B24] Caviness J. N. Driver-Dunckley E. Connor D. J. Sabbagh M. N. Hentz J. G. Noble B. (2007). Defining mild cognitive impairment in Parkinson’s disease. *Mov. Disord.* 22 1272–1277. 17415797 10.1002/mds.21453

[B25] Cerasa A. Gioia M. C. Valentino P. Nistico R. Chiriaco C. Pirritano D. (2013). Computer-assisted cognitive rehabilitation of attention deficits for multiple sclerosis: a randomized trial with fMRI correlates. *Neurorehabil. Neural Repair*. 27, 284–295. 23192417 10.1177/1545968312465194

[B26] Cerasa A. Gioia M. C. Salsone M. Donzuso G. Chiriaco C. Realmuto S. (2014). Neurofunctional correlates of attention rehabilitation in Parkinson’s disease: an explorative study. *Neurol. Sci.* 35 1173–1180. 10.1007/s10072-014-1666-z 24554416

[B27] Chapman S. B. Aslan S. Spence J. S. Hart J. J. Jr. Bartz E. K. Didehbani N. (2015). Neural mechanisms of brain plasticity with complex cognitive training in healthy seniors. *Cereb. Cortex* 25 396–405. 10.1093/cercor/bht234 23985135 PMC4351428

[B28] Cikajlo I. Peterlin Potisk K. (2019). Advantages of using 3D virtual reality based training in persons with Parkinson’s disease: a parallel study. *J. Neuroeng. Rehabil.* 16:119. 10.1186/s12984-019-0601-1 PMC6798369 31623622

[B29] Clare L. Woods R. T. (2004). Cognitive training and cognitive rehabilitation for people with early-stage Alzheimer’s disease: a review. *Neuropsychol. Rehabil.* 14 385–401. 10.1080/09602010443000074

[B30] Collins L. E. Paul N. E. Abbas S. F. Leser C. E. Podurgiel S. J. Galtieri D. J. (2011). Oral tremor induced by galantamine in rats: a model of the parkinsonian side effects of cholinomimetics used to treat Alzheimer’s disease. *Pharmacol. Biochem. Behav.* 99 414–422. 10.1016/j.pbb.2011.05.026 21640750

[B31] Cooper J. A. Sagar H. J. Jordan N. Harvey N. S. Sullivan E. V. (1991). Cognitive impairment in early, untreated Parkinson’s disease and its relationship to motor disability. *Brain* 114(Pt 5) 2095–2122. 10.1093/brain/114.5.2095 1933236

[B32] Costa A. Peppe A. Serafini F. Zabberoni S. Barban F. Caltagirone C. (2014). Prospective memory performance of patients with Parkinson’s disease depends on shifting aptitude: evidence from cognitive rehabilitation. *J. Int. Neuropsychol. Soc*. 20, 717–726. 24967725 10.1017/S1355617714000563

[B33] Crotty G. F. Schwarzschild M. A. (2020). Chasing protection in Parkinson’s disease: does exercise reduce risk and progression? *Front. Aging Neurosci.* 12:186. 10.3389/fnagi.2020.00186 32636740 PMC7318912

[B34] Di Filippo M. Picconi B. Tantucci M. Ghiglieri V. Bagetta V. Sgobio C. (2009). Short-term and long-term plasticity at corticostriatal synapses: implications for learning and memory. *Behav. Brain Res.* 199 108–118. 10.1016/j.bbr.2008.09.025 18948145

[B35] Díez-Cirarda M. Ojeda N. Peña J. Cabrera-Zubizarreta A. Lucas-Jiménez O. Gómez-Esteban J. C. (2018). Long-term effects of cognitive rehabilitation on brain, functional outcome and cognition in Parkinson’s disease. *Eur. J. Neurol.* 25 5–12. 10.1111/ene.13472 28940855 PMC5765471

[B36] Doruk D. Gray Z. Bravo G. L. Pascual-Leone A. Fregni F. (2014). Effects of tdcs on executive function in Parkinson’s disease. *Neurosci. Lett.* 582 27–31. 10.1016/j.neulet.2014.08.043 25179996

[B37] Duncan G. W. Khoo T. K. Yarnall A. J. O’brien J. T. Coleman S. Y. Brooks D. J. (2014). Health-related quality of life in early Parkinson’s disease: the impact of nonmotor symptoms. *Mov. Disord.* 29 195–202. 10.1002/mds.25664 24123307

[B38] Edwards J. D. Hauser R. A. O’connor M. L. Valdes E. G. Zesiewicz T. A. Uc E. Y. (2013). Randomized trial of cognitive speed of processing training in Parkinson disease. *Neurology* 81 1284–1290. 10.1212/wnl.0b013e3182a823ba 24014503 PMC3806923

[B39] Elgh E. Domellof M. Linder J. Edstrom M. Stenlund H. Forsgren L. (2009). Cognitive function in early Parkinson’s disease: a population-based study. *Eur. J. Neurol.* 16 1278–1284. 10.1111/j.1468-1331.2009.02707.x 19538208

[B40] Emre M. Aarsland D. Brown R. Burn D. J. Duyckaerts C. Mizuno Y. (2007). Clinical diagnostic criteria for dementia associated with Parkinson’s disease. *Mov. Disord.* 22 1689–1707. 17542011 10.1002/mds.21507

[B41] Emre M. Poewe W. De Deyn P. P. Barone P. Kulisevsky J. Pourcher E. (2014). Long-term safety of rivastigmine in parkinson disease dementia: an open-label, randomized study. *Clin. Neuropharmacol.* 37 9–16. 10.1097/wnf.0000000000000010 24434526

[B42] Fellman D. Salmi J. Ritakallio L. Ellfolk U. Rinne J. O. Laine M. (2020). Training working memory updating in Parkinson’s disease: a randomised controlled trial. *Neuropsychol. Rehabil.* 30 673–708. 10.1080/09602011.2018.1489860 29968519

[B43] Folkerts A.-K. Dorn M. E. Roheger M. Maassen M. Koerts J. Tucha O. (2018). Cognitive stimulation for individuals with Parkinson’s disease dementia living in long-term care: preliminary data from a randomized crossover pilot study. *Parkinsons Dis.* 2018:8104673. 10.1155/2018/8104673 PMC6304852 30631420

[B44] Foltynie T. Brayne C. E. Robbins T. W. Barker R. A. (2004). The cognitive ability of an incident cohort of Parkinson’s patients in the UK. The Campaign study. *Brain* 127 550–560. 10.1093/brain/awh067 14691062

[B45] Ge S. Zhu Z. Wu B. Mcconnell E. S. (2018). Technology-based cognitive training and rehabilitation interventions for individuals with mild cognitive impairment: a systematic review. *BMC Geriatr.* 18:213. 10.1186/s12877-018-0893-1 30219036 PMC6139138

[B46] Gomez-Pinilla F. Vaynman S. Ying Z. (2008). Brain-derived neurotrophic factor functions as a metabotrophin to mediate the effects of exercise on cognition. *Eur. J. Neurosci.* 28 2278–2287. 10.1111/j.1460-9568.2008.06524.x 19046371 PMC2805663

[B47] Gurevich T. Y. Shabtai H. Korczyn A. D. Simon E. S. Giladi N. (2006). Effect of rivastigmine on tremor in patients with Parkinson’s disease and dementia. *Mov. Disord.* 21 1663–1666. 10.1002/mds.20971 16941467

[B48] Halliday G. M. Leverenz J. B. Schneider J. S. Adler C. H. (2014). The neurobiological basis of cognitive impairment in Parkinson’s disease. *Mov. Disord.* 29 634–650. 24757112 10.1002/mds.25857 PMC4049032

[B49] Hely M. A. Reid W. G. Adena M. A. Halliday G. M. Morris J. G. (2008). The Sydney multicenter study of Parkinson’s disease: the inevitability of dementia at 20 years. *Mov. Disord.* 23 837–844. 10.1002/mds.21956 18307261

[B50] Hilker R. Thomas A. V. Klein J. C. Weisenbach S. Kalbe E. Burghaus L. (2005). Dementia in Parkinson disease: functional imaging of cholinergic and dopaminergic pathways. *Neurology* 65 1716–1722. 10.1212/01.wnl.0000191154.78131.f6 16344512

[B51] Hindle J. V. Petrelli A. Clare L. Kalbe E. (2013). Nonpharmacological enhancement of cognitive function in Parkinson’s disease: a systematic review. *Mov. Disord.* 28 1034–1049. 10.1002/mds.25377 23426759

[B52] Javadi A. H. Brunec I. K. Walsh V. Penny W. D. Spiers H. J. (2014). Transcranial electrical brain stimulation modulates neuronal tuning curves in perception of numerosity and duration. *Neuroimage* 102(Pt 2) 451–457. 10.1016/j.neuroimage.2014.08.016 25130301 PMC4229383

[B53] Kalbe E. Rehberg S. P. Heber I. Kronenbuerger M. Schulz J. B. Storch A. (2016). Subtypes of mild cognitive impairment in patients with Parkinson’s disease: evidence from the landscape study. *J. Neurol. Neurosurg. Psychiatry* 87 1099–1105. 27401782 10.1136/jnnp-2016-313838

[B54] Kehagia A. A. Barker R. A. Robbins T. W. (2010). Neuropsychological and clinical heterogeneity of cognitive impairment and dementia in patients with Parkinson’s disease. *Lancet Neurol.* 9 1200–1213. 10.1016/s1474-4422(10)70212-x 20880750

[B55] Kehagia A. A. Barker R. A. Robbins T. W. (2013). Cognitive impairment in Parkinson’s disease: the dual syndrome hypothesis. *Neurodegener. Dis.* 11 79–92. 10.1159/000341998 23038420 PMC5079071

[B56] Kulisevsky J. Avila A. Barbanoj M. Antonijoan R. Berthier M. L. Gironell A. (1996). Acute effects of levodopa on neuropsychological performance in stable and fluctuating Parkinson’s disease patients at different levodopa plasma levels. *Brain* 119(Pt 6) 2121–2132. 10.1093/brain/119.6.2121 9010015

[B57] Lampit A. Hallock H. Valenzuela M. (2014). Computerized cognitive training in cognitively healthy older adults: a systematic review and meta-analysis of effect modifiers. *PLoS Med.* 11:e1001756. 10.1371/journal.pmed.1001756 25405755 PMC4236015

[B58] Lawrence B. J. Gasson N. Johnson A. R. Booth L. Loftus A. M. (2018). Cognitive training and transcranial direct current stimulation for mild cognitive impairment in Parkinson’s disease: a randomized controlled trial. *Parkinsons Dis.* 2018:4318475. 10.1155/2018/4318475 PMC5892209 29780572

[B59] Leung I. H. Walton C. C. Hallock H. Lewis S. J. Valenzuela M. Lampit A. (2015). Cognitive training in Parkinson disease: a systematic review and meta-analysis. *Neurology* 85 1843–1851. 10.1212/wnl.0000000000002145 26519540 PMC4662707

[B60] Levin B. E. Katzen H. L. (2005). Early cognitive changes and nondementing behavioral abnormalities in Parkinson’s disease. *Adv. Neurol.* 96 84–94. 16383214

[B61] Lövdén M. Bäckman L. Lindenberger U. Schaefer S. Schmiedek F. (2010). A theoretical framework for the study of adult cognitive plasticity. *Psychol. Bull.* 136 659–676. 10.1037/a0020080 20565172

[B62] Mcdaniel M. A. Guynn M. J. Einstein G. O. Breneiser J. (2004). Cue-focused and reflexive-associative processes in prospective memory retrieval. *J. Exp. Psychol. Learn. Mem. Cogn*. 30, 605–614. 15099129 10.1037/0278-7393.30.3.605

[B63] Maggio M. G. De Cola M. C. Latella D. Maresca G. Finocchiaro C. La Rosa G. (2018). What about the role of virtual reality in Parkinson disease’s cognitive rehabilitation? Preliminary findings from a randomized clinical trial. *J. Geriatr. Psychiatry Neurol.* 31 312–318. 10.1177/0891988718807973 30360679

[B64] Malchow B. Keller K. Hasan A. Dorfler S. Schneider-Axmann T. Hillmer-Vogel U. (2015). Effects of endurance training combined with cognitive remediation on everyday functioning, symptoms, and cognition in multiepisode schizophrenia patients. *Schizophr. Bull.* 41 847–858. 10.1093/schbul/sbv020 25782770 PMC4466186

[B65] Manenti R. Brambilla M. Benussi A. Rosini S. Cobelli C. Ferrari C. (2016). Mild cognitive impairment in Parkinson’s disease is improved by transcranial direct current stimulation combined with physical therapy. *Mov. Disord.* 31 715–724. 10.1002/mds.26561 26880536

[B66] Marsh L. Biglan K. Gerstenhaber M. Williams J. R. (2009). Atomoxetine for the treatment of executive dysfunction in Parkinson’s disease: a pilot open-label study. *Mov. Disord.* 24 277–282. 10.1002/mds.22307 19025777 PMC2683743

[B67] McCain K. R. Sawyer T. S. Spiller H. A. (2007). Evaluation of centrally acting cholinesterase inhibitor exposures in adults. *Ann. Pharmacother.* 41 1632–1637. 10.1345/aph.1k139 17848422

[B68] Medalia A. Freilich B. (2008). The neuropsychological educational approach to cognitive remediation (near) model: practice principles and outcome studies. *Am. J. Psychiatr. Rehabil.* 11 123–143. 10.1080/15487760801963660

[B69] Mirelman A. Maidan I. Deutsch J. E. (2013). Virtual reality and motor imagery: promising tools for assessment and therapy in Parkinson’s disease. *Mov. Disord.* 28 1597–1608. 10.1002/mds.25670 24132848

[B70] Mohammadi A. (2016). Induction of neuroplasticity by transcranial direct current stimulation. *J. Biomed. Phys. Eng.* 6 205–208. 28144588 PMC5219570

[B71] Mohlman J. Chazin D. Georgescu B. (2011). Feasibility and acceptance of a nonpharmacological cognitive remediation intervention for patients with Parkinson disease. *J. Geriatr. Psychiatry Neurol.* 24 91–97. 10.1177/0891988711402350 21546649

[B72] Monastero R. Cicero C. E. Baschi R. Davì M. Luca A. Restivo V. (2018). Mild cognitive impairment in Parkinson’s disease: the Parkinson’s disease cognitive study (PACOS). *J. Neurol.* 265:1050. 10.1007/s00415-018-8800-4 29478221

[B73] Muhammed K. Manohar S. Ben Yehuda M. Chong T. T.-J. Tofaris G. Lennox G. (2016). Reward sensitivity deficits modulated by dopamine are associated with apathy in Parkinson’s disease. *Brain* 139 2706–2721. 10.1093/brain/aww188 27452600 PMC5035817

[B74] Muslimovic D. Post B. Speelman J. D. Schmand B. (2005). Cognitive profile of patients with newly diagnosed Parkinson disease. *Neurology* 65 1239–1245. 10.1212/01.wnl.0000180516.69442.95 16247051

[B75] Nagle A. Riener R. Wolf P. (2015). High user control in game design elements increases compliance and in-game performance in a memory training game. *Front. Psychol.* 6:1774. 10.3389/fpsyg.2015.01774 26635681 PMC4653717

[B76] Naismith S. L. Diamond K. Carter P. E. Norrie L. M. Redoblado-Hodge M. A. Lewis S. J. (2011). Enhancing memory in late-life depression: the effects of a combined psychoeducation and cognitive training program. *Am. J. Geriatr. Psychiatry* 19, 240–248. 20808114 10.1097/JGP.0b013e3181dba587

[B77] Naismith S. L. Mowszowski L. Diamond K. Lewis S. J. G. (2013). Improving memory in Parkinson’s disease: a healthy brain ageing cognitive training program. *Mov. Disord.* 28 1097–1103. 10.1002/mds.25457 23630134

[B78] Nombela C. Bustillo P. J. Castell P. F. Sanchez L. Medina V. Herrero M. T. (2011). Cognitive rehabilitation in Parkinson’s disease: evidence from neuroimaging. *Front. Neurol*. 2:82. 10.3389/fneur.2011.00082 PMC3244758 22203816

[B79] Nes Begnum M. E. (2010). “Challenges for norwegian PC-users with Parkinson’s disease–a Survey,” in *Lecture Notes in Computer Science*, eds Miesenberger K. Klaus J. Zagler W. Karshmer A. (Heidelberg: Springer).

[B80] Nuechterlein K. H. Ventura J. Mcewen S. C. Gretchen-Doorly D. Vinogradov S. Subotnik K. L. (2016). Enhancing cognitive training through aerobic exercise after a first schizophrenia episode: theoretical conception and pilot study. *Schizophr. Bull.* 42 Suppl 1 S44–S52. 27460618 10.1093/schbul/sbw007 PMC4960434

[B81] Oertel-Knochel V. Mehler P. Thiel C. Steinbrecher K. Malchow B. Tesky V. (2014). Effects of aerobic exercise on cognitive performance and individual psychopathology in depressive and schizophrenia patients. *Eur. Arch. Psychiatry Clin. Neurosci.* 264 589–604. 10.1007/s00406-014-0485-9 24487666

[B82] Orgeta V. Mcdonald K. R. Poliakoff E. Hindle J. V. Clare L. Leroi I. (2020). Cognitive training interventions for dementia and mild cognitive impairment in Parkinson’s disease. *Cochrane Database Syst. Rev.* 2:CD011961. 10.1002/14651858.CD011961.pub2 PMC7043362 32101639

[B83] Owen A. M. James M. Leigh P. N. Summers B. A. Marsden C. D. Quinn N. P. (1992). Fronto-striatal cognitive deficits at different stages of Parkinson’s disease. *Brain* 115(Pt 6) 1727–1751. 10.1093/brain/115.6.1727 1486458

[B84] Pagonabarraga J. Kulisevsky J. (2012). Cognitive impairment and dementia in Parkinson’s disease. *Neurobiol. Dis.* 46 590–596. 22484304 10.1016/j.nbd.2012.03.029

[B85] París A. P. Saleta H. G. De La Cruz Crespo Maraver M. Silvestre E. Freixa M. G. Torrellas C. P. (2011). Blind randomized controlled study of the efficacy of cognitive training in Parkinson’s disease. *Mov. Disord.* 26 1251–1258. 10.1002/mds.23688 21442659

[B86] Pena J. Ibarretxe-Bilbao N. Garcia-Gorostiaga I. Gomez-Beldarrain M. A. Diez-Cirarda M. Ojeda N. (2014). Improving functional disability and cognition in Parkinson disease: randomized controlled trial. *Neurology* 83 2167–2174. 10.1212/wnl.0000000000001043 25361785 PMC4276404

[B87] Pereira J. B. Junque C. Bartres-Faz D. Marti M. J. Sala-Llonch R. Compta Y. (2013). Modulation of verbal fluency networks by transcranial direct current stimulation (TDCS) in Parkinson’s disease. *Brain Stimul.* 6 16–24. 10.1016/j.brs.2012.01.006 22410476

[B88] Pessoa T. M. Coutinho D. S. Pereira V. M. Ribeiro N. P. D. O. Nardi A. E. Silva A. C. D. O. E. (2014). The nintendo wii as a tool for neurocognitive rehabilitation, training and health promotion. *Comput. Hum. Behav.* 31 384–392. 10.1016/j.chb.2013.10.025

[B89] Petrelli A. Kaesberg S. Barbe M. T. Timmermann L. Fink G. R. Kessler J. (2014). Effects of cognitive training in Parkinson’s disease: a randomized controlled trial. *Parkinsonism Relat. Disord.* 20 1196–1202. 25242806 10.1016/j.parkreldis.2014.08.023

[B90] Petrelli A. Kaesberg S. Barbe M. T. Timmermann L. Rosen J. B. Fink G. R. (2015). Cognitive training in Parkinson’s disease reduces cognitive decline in the long term. *Eur. J. Neurol.* 22 640–647. 10.1111/ene.12621 25534579

[B91] Pompeu J. E. Mendes F. A. Silva K. G. Lobo A. M. Oliveira Tde P. Zomignani A. P. (2012). Effect of nintendo wii-based motor and cognitive training on activities of daily living in patients with Parkinson’s disease: a randomised clinical trial. *Physiotherapy* 98 196–204. 10.1016/j.physio.2012.06.004 22898575

[B92] Rahe J. Liesk J. Rosen J. B. Petrelli A. Kaesberg S. Onur O. A. (2015). Sex differences in cognitive training effects of patients with amnestic mild cognitive impairment. *Neuropsychol. Dev. Cogn. B Aging. Neuropsychol. Cogn.* 22 620–638. 10.1080/13825585.2015.1028883 25818876

[B93] Reuter I. Mehnert S. Sammer G. Oechsner M. Engelhardt M. (2012). Efficacy of a multimodal cognitive rehabilitation including psychomotor and endurance training in Parkinson’s disease. *J. Aging Res.* 2012:235765. 10.1155/2012/235765 PMC3447352 23008772

[B94] Rolinski M. Fox C. Maidment I. Mcshane R. (2012). Cholinesterase inhibitors for dementia with Lewy bodies, Parkinson’s disease dementia and cognitive impairment in Parkinson’s disease. *Cochrane Database Syst. Rev.* 2012:CD006504. 10.1002/14651858.CD006504.pub2 PMC8985413 22419314

[B95] Sammer G. Reuter I. Hullmann K. Kaps M. Vaitl D. (2006). Training of executive functions in Parkinson’s disease. *J. Neurol. Sci*. 248, 115–119. 16765378 10.1016/j.jns.2006.05.028

[B96] Sanchez P. Pena J. Bengoetxea E. Ojeda N. Elizagarate E. Ezcurra J. (2014). Improvements in negative symptoms and functional outcome after a new generation cognitive remediation program: a randomized controlled trial. *Schizophr. Bull.* 40 707–715. 10.1093/schbul/sbt057 23686130 PMC3984510

[B97] Saredakis D. Collins-Praino L. E. Gutteridge D. S. Stephan B. C. M. Keage H. A. D. (2019). Conversion to MCI and dementia in Parkinson’s disease: a systematic review and meta-analysis. *Park. Relat. Disord.* 65 20–31. 10.1016/j.parkreldis.2019.04.020 31109727

[B98] Seppi K. Weintraub D. Coelho M. Perez-Lloret S. Fox S. H. Katzenschlager R. (2011). The movement disorder society evidence-based medicine review update: treatments for the non-motor symptoms of Parkinson’s disease. *Mov. Disord.* 26(Suppl. 3) S42–S80. 22021174 10.1002/mds.23884 PMC4020145

[B99] Sinforiani E. Banchieri L. Zucchella C. Pacchetti C. Sandrini G. (2004). Cognitive rehabilitation in Parkinson’s disease. *Arch. Gerontol. Geriatr.* 38 387–391. 10.1016/j.archger.2004.04.049 15207437

[B100] Soleimani M. A. Negarandeh R. Bastani F. Greysen R. (2014). Disrupted social connectedness in people with Parkinson’s disease. *Br. J. Community Nurs.* 19 136–141. 10.12968/bjcn.2014.19.3.136 24897835

[B101] Song I. U. Kim J. S. Ryu S. Y. Lee S. B. An J. Y. Lee K. S. (2008). Donepezil-induced jaw tremor. *Park. Relat. Disord.* 14 584–585. 10.1016/j.parkreldis.2008.01.003 18321753

[B102] Song S. K. Yoshino J. Le T. Q. Lin S. J. Sun S. W. Cross A. H. (2005). Demyelination increases radial diffusivity in corpus callosum of mouse brain. *Neuroimage* 26 132–140. 10.1016/j.neuroimage.2005.01.028 15862213

[B103] Stoetzner C. R. Pettibone J. R. Berke J. D. (2010). State-dependent plasticity of the corticostriatal pathway. *Neuroscience* 165 1013–1018. 10.1016/j.neuroscience.2009.11.031 19932155 PMC2814943

[B104] Tarraga L. Boada M. Modinos G. Espinosa A. Diego S. Morera A. (2006). A randomised pilot study to assess the efficacy of an interactive, multimedia tool of cognitive stimulation in Alzheimer’s disease. *J. Neurol. Neurosurg. Psychiatry* 77, 1116–1121. 16820420 10.1136/jnnp.2005.086074 PMC2077529

[B105] Talboom J. S. West S. G. Engler-Chiurazzi E. B. Enders C. K. Crain I. Bimonte-Nelson H. A. (2014). Learning to remember: cognitive training-induced attenuation of age-related memory decline depends on sex and cognitive demand, and can transfer to untrained cognitive domains. *Neurobiol. Aging* 35 2791–2802. 10.1016/j.neurobiolaging.2014.06.008 25104561 PMC4252709

[B106] Thomas M. Lenka A. Kumar Pal P. (2017). Handwriting analysis in Parkinson’s disease: current status and future directions. *Mov. Disord. Clin. Pract.* 4 806–818. 10.1002/mdc3.12552 30363367 PMC6174397

[B107] Valkanova V. Eguia Rodriguez R. Ebmeier K. P. (2014). Mind over matter–what do we know about neuroplasticity in adults? *Int. Psychogeriatr.* 26 891–909. 10.1017/s1041610213002482 24382194

[B108] Van De Weijer S. C. Kuijf M. L. De Vries N. M. Bloem B. R. Duits A. A. (2019). Do-it-yourself gamified cognitive training: viewpoint. *JMIR Serious Games* 7:e12130. 10.2196/12130 31066713 PMC6528436

[B109] van de Weijer S. C. F. Duits A. A. Bloem B. R. De Vries N. M. Kessels R. P. C. Köhler S. (2020). Feasibility of a cognitive training game in Parkinson’s disease: the randomized parkin’play study. *Eur. Neurol.* 83 426–432. 10.1159/000509685 32756067 PMC7592931

[B110] Vlagsma T. T. Duits A. A. Dijkstra H. T. Van Laar T. Spikman J. M. (2020). Effectiveness of reset; a strategic executive treatment for executive dysfunctioning in patients with Parkinson’s disease. *Neuropsychol. Rehabil.* 30 67–84. 29566588 10.1080/09602011.2018.1452761

[B111] Walton C. C. Naismith S. L. Lampit A. Mowszowski L. Lewis S. J. G. (2017). Cognitive training in Parkinson’s disease. *Neurorehabil. Neural Repair* 31 207–216. 27899737 10.1177/1545968316680489

[B112] Wang H. F. Yu J. T. Tang S. W. Jiang T. Tan C. C. Meng X. F. (2015). Efficacy and safety of cholinesterase inhibitors and memantine in cognitive impairment in Parkinson’s disease, Parkinson’s disease dementia, and dementia with Lewy bodies: systematic review with meta-analysis and trial sequential analysis. *J. Neurol. Neurosurg. Psychiatry* 86 135–143. 10.1136/jnnp-2014-307659 24828899

[B113] Weintraub D. Mavandadi S. Mamikonyan E. Siderowf A. D. Duda J. E. Hurtig H. I. (2010). Atomoxetine for depression and other neuropsychiatric symptoms in Parkinson disease. *Neurology* 75 448–455. 10.1212/wnl.0b013e3181ebdd79 20679638 PMC2918470

[B114] Williams-Gray C. H. Evans J. R. Goris A. Foltynie T. Ban M. Robbins T. W. (2009). The distinct cognitive syndromes of Parkinson’s disease: 5 year follow-up of the campaign cohort. *Brain* 132 2958–2969. 10.1093/brain/awp245 19812213

[B115] Williams-Gray C. H. Foltynie T. Brayne C. E. Robbins T. W. Barker R. A. (2007). Evolution of cognitive dysfunction in an incident Parkinson’s disease cohort. *Brain* 130 1787–1798. 10.1093/brain/awm111 17535834

[B116] Wolinsky F. D. Vander Weg M. W. Martin R. Unverzagt F. W. Ball K. K. Jones R. N. (2009). The effect of speed-of-processing training on depressive symptoms in active. *J. Gerontol. A Biol. Sci. Med. Sci.* 64 468–472. 10.1093/gerona/gln044 19181719 PMC2657170

[B117] Xu J. Zhang J. Wang J. Li G. Hu Q. Zhang Y. (2016). Abnormal fronto-striatal functional connectivity in Parkinson’s disease. *Neurosci. Lett.* 613 66–71. 10.1016/j.neulet.2015.12.041 26724369

[B118] Zimmermann R. Gschwandtner U. Benz N. Hatz F. Schindler C. Taub E. (2014). Cognitive training in Parkinson disease: cognition-specific vs nonspecific computer training. *Neurology* 82 1219–1226. 10.1212/wnl.0000000000000287 24623840

